# Characterizing ATP processing by the AAA+ protein p97 at the atomic level

**DOI:** 10.1038/s41557-024-01440-0

**Published:** 2024-02-07

**Authors:** Mikhail Shein, Manuel Hitzenberger, Tat Cheung Cheng, Smruti R. Rout, Kira D. Leitl, Yusuke Sato, Martin Zacharias, Eri Sakata, Anne K. Schütz

**Affiliations:** 1 Faculty for Chemistry and Pharmacy, Ludwig-Maximilians-Universität München, München, Germany; 2 Bavarian NMR Center, Technical University of Munich, Garching, Germany; 3 Institute of Structural Biology, Helmholtz Zentrum München, Neuherberg, Germany; 4 Physics Department and Center of Protein Assemblies, Technical University of Munich, Garching, Germany; 5 Institute for Neuropathology, University Medical Center Göttingen, Göttingen, Germany; 6 Multiscale Bioimaging: from Molecular Machines to Networks of Excitable Cells (MBExC), University of Göttingen, Göttingen, Germany; 7 Institute for Auditory Neuroscience, University Medical Center Göttingen, Göttingen, Germany; 8 Center for Research on Green Sustainable Chemistry, Graduate School of Engineering, Tottori University, Tottori, Japan; 9 Department of Chemistry and Biotechnology, Graduate School of Engineering, Tottori University, Tottori, Japan

**Keywords:** Computational biophysics, Cryoelectron microscopy, NMR spectroscopy

## Abstract

The human enzyme p97 regulates various cellular pathways by unfolding hundreds of protein substrates in an ATP-dependent manner, making it an essential component of protein homeostasis and an impactful pharmacological target. The hexameric complex undergoes substantial conformational changes throughout its catalytic cycle. Here we elucidate the molecular motions that occur at the active site in the temporal window immediately before and after ATP hydrolysis by merging cryo-EM, NMR spectroscopy and molecular dynamics simulations. p97 populates a metastable reaction intermediate, the ADP·P_i_ state, which is poised between hydrolysis and product release. Detailed snapshots reveal that the active site is finely tuned to trap and eventually discharge the cleaved phosphate. Signalling pathways originating at the active site coordinate the action of the hexamer subunits and couple hydrolysis with allosteric conformational changes. Our multidisciplinary approach enables a glimpse into the sophisticated spatial and temporal orchestration of ATP handling by a prototype AAA+ protein.

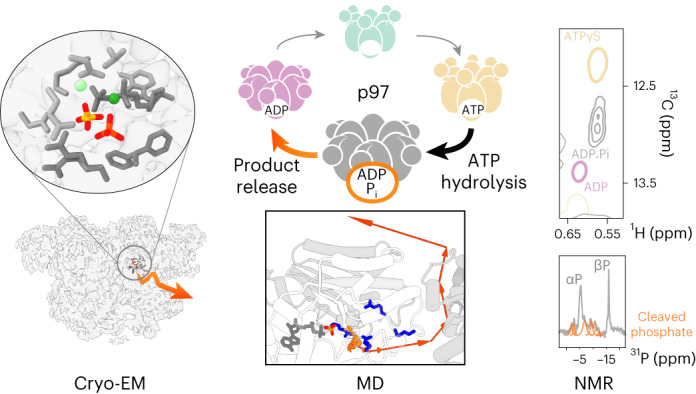

## Abstract (web summary)

The human ATP-hydrolysing enzyme p97 populates a metastable reaction intermediate, the ADP·P_i_ state, which is poised between hydrolysis and product release. Now, molecular motions at the active site in the temporal window immediately before and after ATP hydrolysis have been elucidated by merging cryo-EM, NMR spectroscopy and molecular dynamics simulations.

## Main

**Fig. 1:**
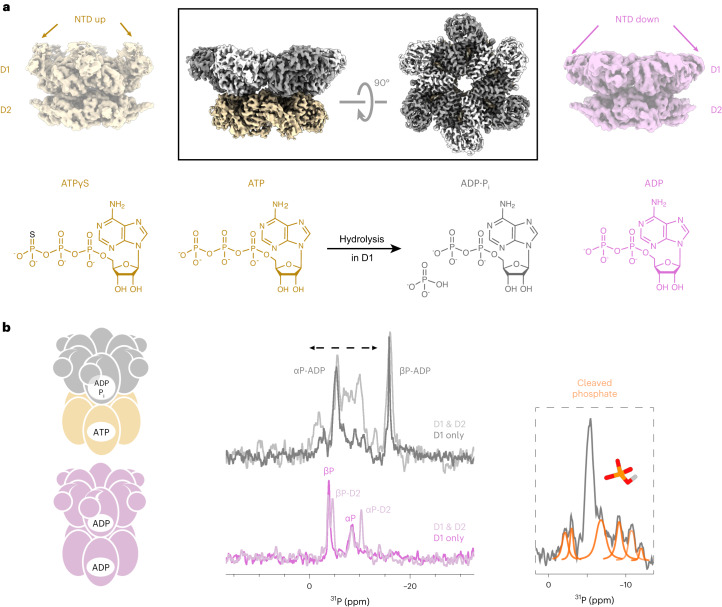
Global conformational changes linked to nucleotide turnover in the tandem ATPase p97. **a**, Box: single-particle cryo-EM reconstruction of p97 in the ADP·P_i_ state reveals a symmetric hexamer with NTD domains in the ‘down’ position. For comparison, cryo-EM maps of ATPγS- and ADP-bound p97 are shown (EMDB 3298 and 3299)^5^. The colouring reflects the bound nucleotides shown underneath. Details on reconstruction are provided in Supplementary Figs. 1–3. **b**, Left and middle: magic-angle spinning ^1^H → ^31^P cross-polarization NMR spectra of p97-bound nucleotide in the presence of ATP (top) and ADP (bottom). The p97-ND1L hexamer (residues 1–480) is ATPase active^6^ and contains only signals from D1; the corresponding spectra of full-length p97 are shown in lighter hues. Right: the observation of multiple weaker signals (orange fit) is ascribed to phosphate ions in chemically distinct environments. These signals must derive from the cleaved γ-phosphate of ATP locked in the D1 active site, because thio-substitution at this position in ATP results in a strong downfield shift^11^.

The ATP-dependent enzyme p97 powers diverse energy-consuming processes in the cell^1^, including proteasomal degradation^2^, membrane fusion^3^ and autophagy^4^. p97 is a homo-hexamer, in which each subunit comprises two ATPase domains, D1 and D2, that assemble into two stacked rings (Fig. 1a). Its N-terminal domain (NTD) recruits cofactors and substrates and is positioned according to the nucleotide bound in D1: elevated above the D1 ring when ATP is bound (NTD ‘up’) and coplanar in the ADP-bound form (NTD ‘down’)^5,6^. As a result, the NTD undergoes a large-scale motion during the ATP-hydrolysis cycle^5^. The p97 hexamer is symmetric, with coherent positions of the six NTDs, in the absence of substrates, but it adopts an asymmetric staircase conformation when cofactors and substrates are present^7–9^. p97 is a member of the ATPases associated with diverse cellular activities (AAA+) superfamily, which features conserved functional elements for nucleotide binding and hydrolysis, such as the Walker A and B motifs, the arginine finger and the sensor motifs^10^. As the p97 hexamer assembles, 12 active sites emerge at the inter-subunit interfaces, allowing for allosteric coordination of enzymatic activity among the subunits^10^.

We previously reported that, in the presence of ATP and the absence of cofactors and substrates, p97 populates a uniform nucleotide state, where D1 is occupied with ADP and still hosts the cleaved phosphate (P_i_) ion^11^. Conformational analysis by nuclear magnetic resonance (NMR) spectroscopy indicated that the observed state is distinct from apo, ADP- or slowly hydrolysable ATPγS states. A reaction intermediate in which the bond between the γ- and β-phosphate groups of ATP has been cleaved but neither reaction product released was postulated 50 years ago and termed the ‘ADP·P_i_’ state^12^. Molecular dynamics (MD) simulations could capture this state at the atomic level^13–16^, yet it has been refractory to experimental characterization owing to its limited lifetime.

Single-particle cryo-electron microscopy (cryo-EM) enables the structural analysis of such transient species, provided they are successfully captured during the plunge-freezing process^17,18^. Resolutions below 4 Å are sufficient to establish the identity of the nucleotide^5,19–21^, that is, whether ATP or ADP is bound. However, it remains challenging to determine the location of the cleaved P_i_ ion as its distance to the nucleotide is not known a priori and its location may fluctuate, causing smearing of the cryo-EM density. Exceptions are Hsp70^22^, F-actin^23^, myosin^24^ and F_1_-ATPase^25^, which all form stable ADP·P_i_ adducts with exogenous P_i_ ions that may not reflect the authentic reaction intermediates preceded by enzymatic hydrolysis events. So far, no transient ADP·P_i_ structure after P_i_ cleavage has been reported or recognized as such.

In this Article, we derive the structure of ADP·P_i_-bound p97 via cryo-EM and MD. This snapshot of ATP processing reveals how the active site first accommodates and then releases the cleaved P_i_ ion. We dissect the contributions of active-site residues and identify the underlying triggers that induce domain motion upon hydrolysis. Additionally, we map pathways that coordinate activity between adjacent subunits. Our investigation sheds light on the structural transitions and dynamical changes that accompany ATP processing by multimeric enzymes.

## Results

### Observation of a post-ATP-hydrolysis reaction intermediate

**Extended Data Fig. 1:**
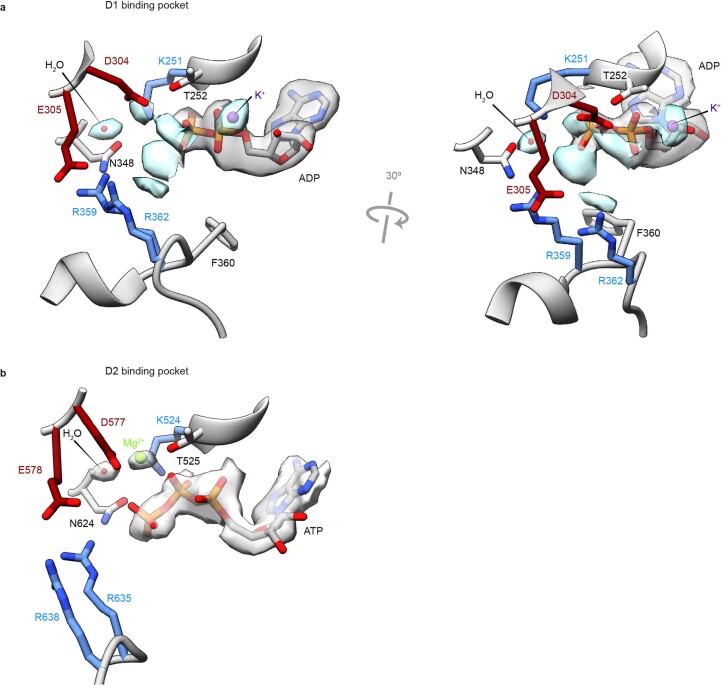
Nucleotides bound in D1 and D2. Zoom of the cryo-EM density that is assigned to the nucleotide bound to full-length p97 in the presence of ATP. **a** and **b**, An ADP molecule with two phosphate groups and an ATP molecule with three phosphate groups are clearly resolved in the binding pockets of D1 and D2 domains, respectively. In MD simulations, Na^+^ or K^+^ ions are often found binding to the α-P of ADP in D1, coinciding with unassigned density in this area. The water molecules in D1 and D2 were identified by the ‘Find Waters’ routine in Coot. Density in the D1 pocket that is marked in cyan remains unidentified by this routine and cannot be explained based on the protein structure either, even if potential side chain rotamers are considered. The presence of discrete and unexplained densities suggests that a distinct chemical environment is captured in the D1 pocket. The densities in panel **a** are shown at threshold levels of 0.0065. The ATP density in panel **b** is shown at a threshold level of 0.0056, while that of Mg^2+^ and H_2_O is shown at 0.0066.

Full-length p97 at physiological Mg^2+^ ion and ATP concentrations in the presence of an ATP-regeneration system was flash-frozen and subjected to single-particle cryo-EM. In agreement with previous cryo-EM studies of p97^26,27^, a mixture of single- and double-ring hexamers was observed. Initial three-dimensional (3D) classification without imposing symmetry revealed that both single and double rings have all six NTDs positioned coplanar with the D1 ring, in the ‘down’ state. Further processing of the double-ring particles with *C*6 symmetry pushed the final resolution to 2.61 Å (Fig. 1a, middle). Overall, the structure of the D1 domain is similar to that of ADP-bound p97^5^. Elements related to the NTD ‘down’ state are fully built, notably the helix-loop conversion in the NTD-D1 linker and NTD-D1 interfaces. The nucleotides in D1 and D2 were assigned to ADP and ATP, respectively (Extended Data Fig. 1). Although the D2 ATP molecule is clearly defined, weak cryo-EM densities are observed around the ADP molecule in D1, hinting at the presence of additional molecules and structural heterogeneity. These densities could potentially arise from water molecules, mono- and divalent ions (Na^+^, K^+^, Mg^2+^ from the buffer), cleaved P_i_ ions or side-chain rotamers of the enzyme.

To confirm the identity of the p97-bound nucleotide, we acquired ^31^P NMR spectra of nucleotide bound to p97 during ATP turnover (Fig. 1b). Comparing the spectra acquired on full-length p97 to a variant lacking the D2 domain (p97-ND1L, residues 1–480^6^), the α-phosphate (P) and β-P signals of the ADP molecule in D1 can be assigned. In addition, multiple weaker signals are attributed to P_i_ ions trapped at the active site in a heterogeneous environment. Electron microscopy and NMR concur that a metastable ADP·P_i_ nucleotide state has been captured in D1, which we subjected to in-depth structural analysis.

### Structure of the active site in the ADP·P_i_ state

**Fig. 2:**
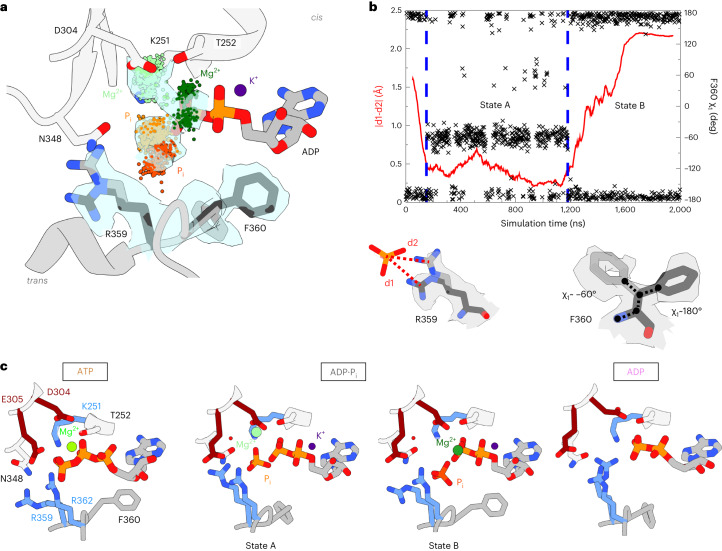
Coordination of the cleaved P_i_ ion in D1. **a**, Zoom-in on the unaccounted densities at the D1 active site. Snapshots from the MD trajectory evaluated at 2-ns intervals identify at least two locations each for P_i_ and Mg^2+^. The convergence between MD and cryo-EM enables the assignment of Mg^2+^ (light for state A, dark green for state B), cleaved P_i_ (orange for state A, orange-red for state B) and the R359/F360 rotamers (light grey for state A, dark grey for state B). The iterative modelling process is outlined in Extended Data Fig. 3. Density threshold levels: Mg^2+^, P_i_ and F360: 0.0056; R359: 0.0062. **b**, Top: in MD simulations of the ADP·P_i_ state, R359 and F360 undergo a correlated motion on a microsecond timescale, evidenced by fluctuations of the side-chain dihedral angle (F360 *χ*_1_) and the phosphate–arginine binding geometry, represented by the distances d1 and d2 between R359-Nη1/Nη2 and the cleaved P_i_ ion. Residual densities at the D1 active site after assignment of the protein and ADP. A transition between the two stable geometries, states A and B, occurs here after ~1,200 ns. Bottom: the side-chain rotamers are visible in the experimental cryo-EM density. **c**, Juxtaposition of the D1 nucleotide binding pocket in ATPγS (PDB 7LMY)^7^, ADP (PDB 5FTK)^5^ and ADP·P_i_ states (PDB 8OOI, this work). Supplementary Fig. 4 highlights the D1 binding pocket from a different orientation and illustrates the distances of key interactions for the P_i_ and Mg^2+^ ions. Source data

The cryo-EM density in D1 revealed an ADP molecule surrounded by multiple unexplained patches of density, extending from the β-P (Fig. 2a and Extended Data Fig. 1a) and close to the arginine finger R359. To ascertain the chemical identity of these densities, we obtained a trajectory of the P_i_ and Mg^2+^ ions immediately after ATP hydrolysis from MD simulations. Starting from ATP-bound p97 hexamer, the ATP molecule in one of the six subunits was converted to ADP·P_i_ in silico, followed by 2 µs of unrestrained simulation. After rearrangements at the active site within the first few nanoseconds, two clusters emerge, indicating stable positions of the Mg^2+^ and P_i_ ions. (1) In the first cluster, termed state A, the leaving P_i_ ion is stabilized by Walker A residue K251 as well as sensor residue N348. R359 binds to P_i_ but sometimes dissociates or binds via water. It is much more mobile than N348, which maintains a persistent binding mode with respect to the P_i_ ion. (2) In the second cluster, termed state B, the leaving P_i_ is detached from K251 and positioned closer to R359 and R362, thus being pulled towards the adjacent, *trans*-acting subunit. These two clusters superimpose well with the unassigned cryo-EM densities (Fig. 2a).

**Extended Data Fig. 2:**
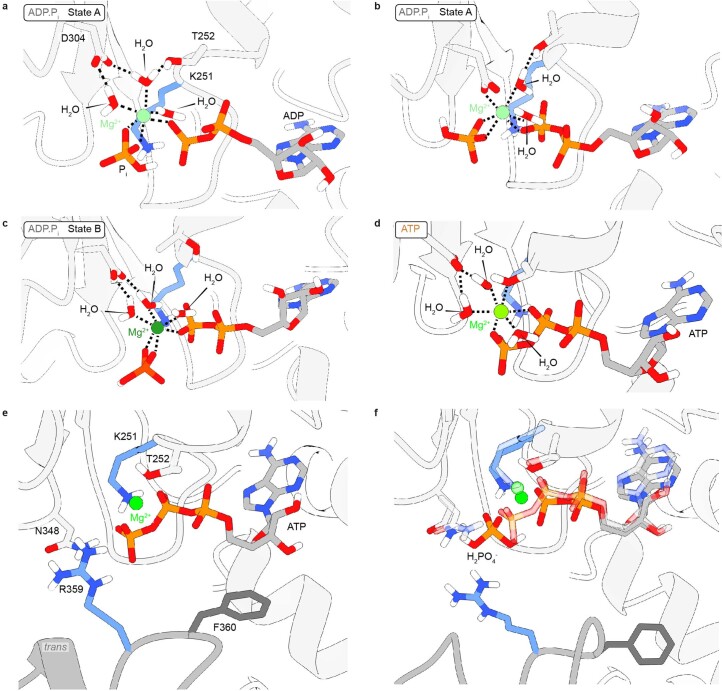
Snapshots from MD simulations of D1 in different nucleotide states. **a-d**, Octahedral coordination of the Mg^2+^ ion in ADP·P_i_ states A **(a, b)** and B **(c)** and in ATP state **(d)** in different MD simulations, which are listed in Supplementary Table 5. For state A, two coordination modes are stable on a microsecond timescale in MD: (**a**) simulations started from the ATP state cleaved *in silico* into ADP and P_i_, where a water molecule stays put between the Mg^2+^ ion and the side chain of D304; (**b**) simulations started from state A of the ADP·P_i_ cryo-EM structure, where the Mg^2+^ ion is coordinated by D304 side chain directly without a bridging water molecule. It is possible that the bridging water molecule exists directly after ATP hydrolysis, in the ‘early’ ADP·P_i_ state, to be replaced by the Mg^2+^ itself in the ‘late’ ADP·P_i_ state, which is captured by cryo-EM. **e**, The X-ray structure of ATPγS-bound p97^29^ was used as a starting structure for MD simulations with ATPγS converted to ATP, of which the first snapshot is shown here. **f**, Snapshot from an MD simulation of the D1 active site containing ADP and a doubly protonated phosphate ion (H_2_PO_4_^−^) superimposed on the ATP state from panel e in transparent. The ADP·P_i_ state with H_2_PO_4_^−^ shows high similarity to ATP-bound state and does not coincide with the experimental cryo-EM density.

In silico analysis suggests that the Mg^2+^ ion stabilizes the leaving P_i_, compensating the coulombic repulsion from the β-P of ADP. In both states, an octahedral coordination geometry of the Mg^2+^ ion is achieved (Extended Data Fig. 2a–c). Compared to the ATP state (Extended Data Fig. 2d), the Mg^2+^ ion dissociates from T252, and the P_i_ ion fills a second coordination site instead. With regard to the protonation state of the leaving P_i_ ion, only the simulation featuring HPO_4_^2−^ is in agreement with the experimental cryo-EM density of the ADP·P_i_ state, whereas the simulation featuring H_2_PO_4_^−^ exhibits conformations and dynamics nearly identical to those of the ATP state (Extended Data Fig. 2e,f).

In our cryo-EM map of the ADP·P_i_ state, we observed distinct rotamers for three residues at the active site: R359 and F360, which interact with the nucleotide in *trans* (Fig. 2b), and H384, which is positioned in the *cis* subunit (Extended Data Fig. 10b, discussed below). In the simulations, the F360 rotamer motion is correlated to the interaction mode between the cleaved phosphate and R359 (Fig. 2b and Supplementary Video 1). The head-on bidentate complex of the P_i_ ion with two amino groups in state A is linked to the F360 *χ*_1_ = −60° conformer, and the lateral monodentate complex of R359 in state B is linked to the *χ*_1_ = 180° conformer.

**Extended Data Fig. 3:**
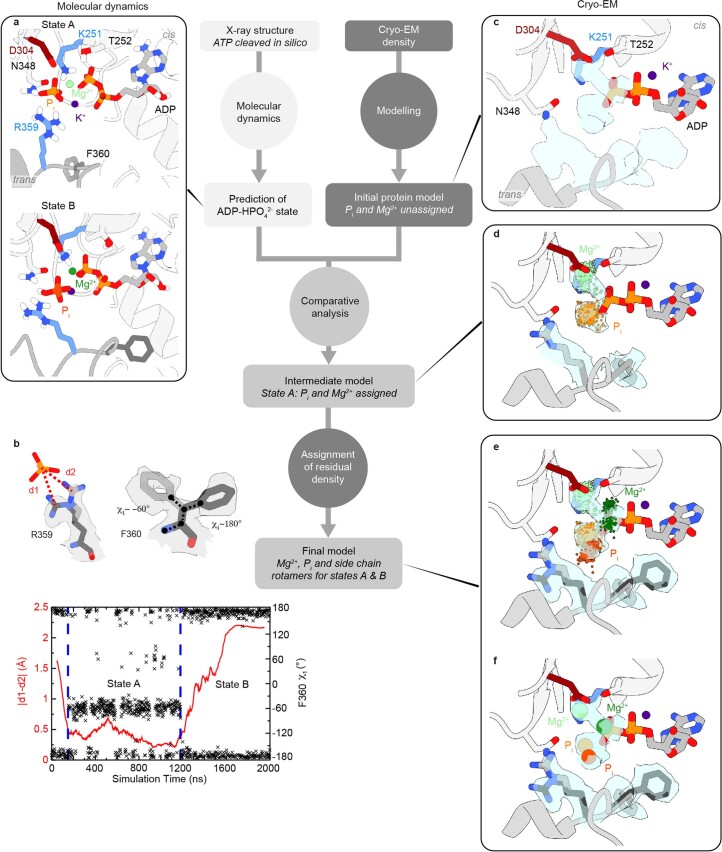
Stepwise assignment of the cryo-EM density at the D1 active site. The positions of the Mg^2+^ and P_i_ ions and the side chain rotamers in ADP·P_i_ state were determined by matching the MD simulations (left column) to the residual cryo-EM density (right column) surrounding the ADP molecule in D1. **a**, Snapshots from simulations of ADP·P_i_ state reveal at least two distinct stable geometries, termed states A and B, which differ in the position of the P_i_ and Mg^2+^ ions. **b**, The side chains of R359 and F360 undergo a correlated motion in the MD trajectory. Left ordinate: the distances d1 and d2 between R359-N^η1^/N^η2^ and the P atom of the cleaved P_i_ ion reflect the side chain conformation of R359. Right ordinate: χ_1_ angle of F360. **c**, Residual densities at the D1 active site after assignment of the protein and ADP. Two side chain rotamers each for R359 and F360 are evident from the cryo-EM density. **d**, Snapshots taken from an MD simulation sampling state A every 2 ns superimposed on the structural model. The predicted positions of the P_i_ (orange) and Mg^2+^ (light green) ions and one set of rotamers for R359 and F360 (light grey) coincide with the unassigned cryo-EM density. **e**, Convergence between MD snapshots of state B and residual densities: P_i_ (orange red) and Mg^2+^ (dark green) ions, second set of rotamers for R359 and F360 (dark grey). **f**, Final structural model of the D1 binding pocket in the ADP·P_i_ state. The densities in panels **c**-**f** are shown at threshold levels of 0.0051–0.0062.

By iterative integration of MD and EM, we determined the positions of the leaving P_i_ and Mg^2+^ ions as well as the associated conformations of active-site residues (Fig. 2a and Extended Data Fig. 3). The following features set apart the ADP·P_i_ state from the ADP and ATPγS states (Fig. 2c and Supplementary Fig. 4): the active site is heterogeneous with at least two distinct positions for P_i_ and Mg^2+^ ions; K251 interacts more with the leaving P_i_ than with ADP; the Mg^2+^ ion has dissociated from T252 to interact with D304; N348 coordinates the P_i_ ion; and R359 and F360 occupy two side-chain rotamer states, reflected in the microsecond-timescale motion in MD simulations.

### Contribution of individual residues to the processing of ATP

**Fig. 3:**
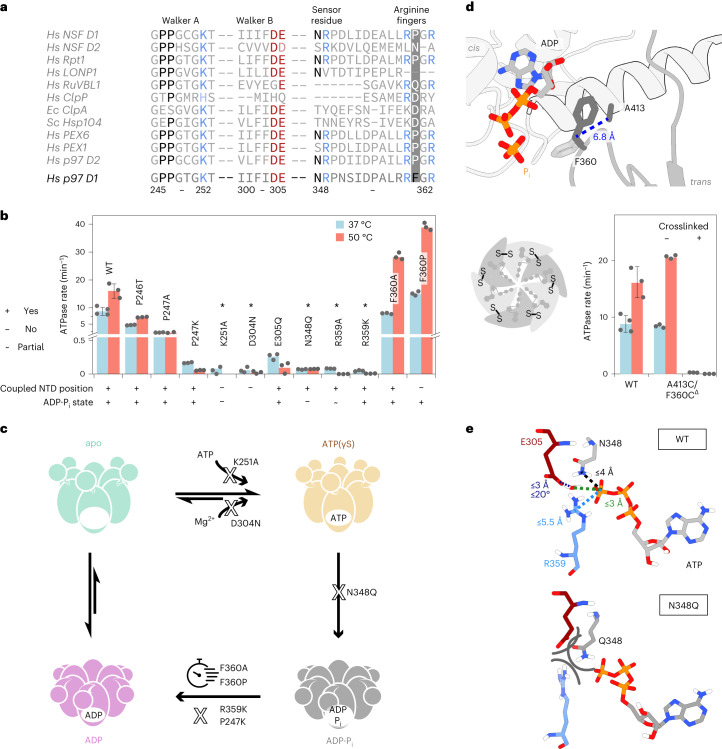
Function of active-site residues in the ATP-hydrolysis cycle. **a**, Sequence alignment of AAA+ family proteins from *Homo sapiens* (*Hs*), *Escherichia coli* (*Ec*) and *Saccharomyces cerevisiae* (*Sc*). Although several key motifs are highly conserved, F360 is unique to the p97 D1 domain (Supplementary Fig. 5). **b**, ATPase rates of p97-ND1L bearing point mutations at the active site and their functional defects deduced from NMR analysis. All presented mutants form hexamers. Only N348Q fully abolishes ATP hydrolysis, and only mutations of F360 have a stimulatory effect on phosphate release. Asterisks designate ATPase inactive mutants. ‘Coupled NTD position’ indicates whether the mutant exhibits the same change in NTD position upon nucleotide binding as wild-type (WT) p97. ‘ADP·P_i_ state’ indicates that this state is observed during ATP turnover. Data are presented as mean values. Error bars represent s.d. for *n* = 4 biologically independent replicates. ATPase rates were determined in *n* = 2–4 replicates, as indicated by the corresponding data points. The ATPase rate of K251A at 50 °C could not be determined due to the low thermal stability. **c**, Impact of mutations on the four steps of the ATP-hydrolysis cycle. **d**, Top: in the ADP·P_i_ state, F360 from the *trans*-acting subunit samples two side-chain rotamer states, one of which contacts helix α_407–423_ of the active subunit. Bottom: crosslinking of C360 to this helix at C413, but not the mutations alone, abolishes ATPase activity of D1. ^Δ^ designates a cysteine-free p97 variant. Data are presented as mean values. Error bars represent s.d. for *n* = 4 biologically independent replicates. ATPase rates were determined in *n* = 3 or 4 replicates, as indicated by the corresponding data points. **e**, Criteria that define hydrolysis-active conformations^15^, amended for p97. (i) A water molecule next to the terminal phosphate (dark green) forms a hydrogen bond to the E305 side chain (dark blue). This lytic water molecule is polarized and thus activated for attack. (ii) R359 polarizes the γ-phosphate and is poised to hydrogen-bond after cleavage (light blue). (iii) The γ-phosphate is held in place by N348 via a hydrogen bond (black). Simulations of the N348Q mutant lack hydrolysis-active conformations due to steric hindrance from the longer Q side chain. Source data

**Extended Data Fig. 4:**
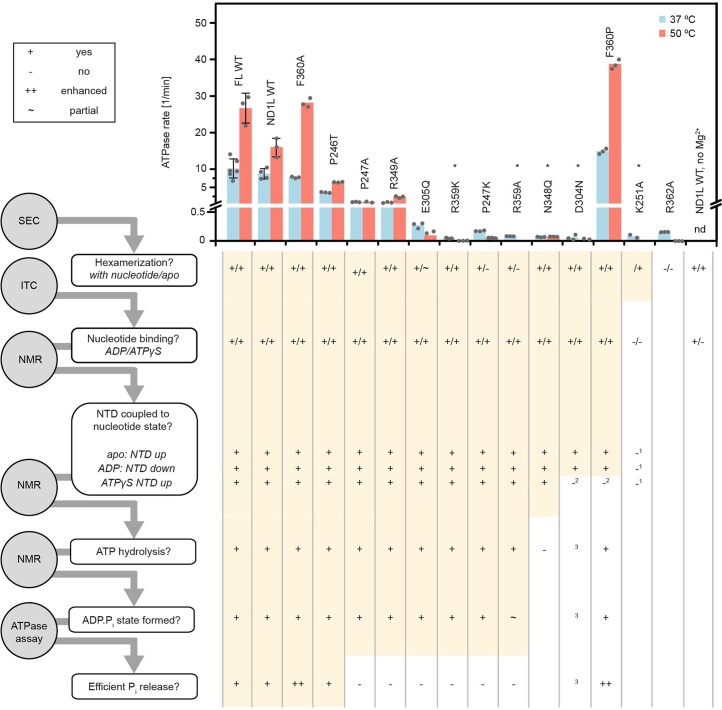
Structural and functional defects of D1-active site mutants. Left: flowchart of biophysical methods to characterize p97-ND1L mutants with respect to their structural and functional integrity. Methods are shown as grey circles: size-exclusion chromatography (SEC), isothermal titration calorimetry (ITC), NMR conformational analysis, ATPase rate measurement; mutant properties are shown as white boxes. Right top: ATPase rates of the mutants. Right bottom: The sequence of assays was pursued as long as a given mutant was assessed positive in the previous category (+, yellow background) but not if it behaved completely (−) or partially (~) different from the wt. Annotations: * Mutant displays no detectable ATPase activity; nd no data; ^1^ spectral change detected in the presence of any nucleotide, irrespective of type, NTD position mixed; ^2^ NTD in ‘down’ state in presence of slowly-hydrolysable ATP analogues; ^3^ ADP·P_i_ state cannot be distinguished from the ATP state by NMR, precluding categorization of the mutant. The corresponding NMR spectra are shown in Extended Data Fig. 5 and Supplementary Fig. 6–9. The nucleotide dissociation constants are listed in Supplementary Table 7. Data are presented as mean values. Error bars represent s.d. for n=3-4 biologically independent replicates. ATPase rates were determined in n=2–4 replicates as indicated by corresponding data points. ATPase rate of K251A at 50 °C could not be determined due to low thermal stability. Source data

**Extended Data Fig. 5:**
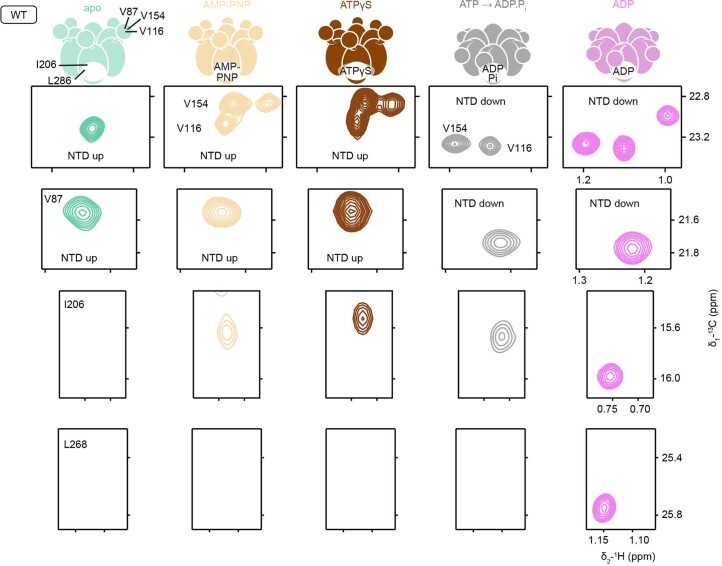
NMR probes indicate global conformation and nucleotide state of p97. Selected spectral regions from HMQC spectra of *proR*-^13^CH_3_-ILVM-labelled p97-ND1L wt acquired at 37 °C (apo) or 50 °C (all others). Residues V116, V154 and V87 report on the NTD position^30^ (‘up’ in apo, AMP-PNP and ATPγS states *vs*. ‘down’ in ADP and ADP·P_i_ states), while residues I206 and L268 report on the conformation of the D1 active site and its bound nucleotide. ATPγS^74^ and AMP-PNP^75^ are slowly hydrolysable analogues of ATP. When a mutant assumes the NTD ‘down’ position in the presence of ATPγS, this can be either due to hydrolysis of ATPγS and slow release of thiophosphate or due to a structural defect that prevents the formation of an NTD ‘up’ state in response to Mg^2+^ and ATPγS binding. Therefore, a spectrum in the presence of Mg^2+^ and AMP-PNP, which features a chemically more stable phosphate linkage, was recorded in addition. Excerpts from the corresponding spectra of point mutants (F360P, D304N, K251A, N348Q) are shown in Supplementary Fig. 6–9.

The D1 domain of p97 contains both signature AAA+ motifs and unique elements (Fig. 3a). To explore the roles of the active-site residues, we conducted biophysical assays on point-mutated p97-ND1L. Each mutant was subjected to a stepwise assessment of defects in assembly, nucleotide binding and ATPase activity (Extended Data Fig. 4). We also studied the conformational dynamics of the mutants in response to the bound nucleotide by NMR (Extended Data Fig. 5).

The results are summarized in Fig. 3b,c. In brief, all mutants except K251A (Supplementary Fig. 6 and Supplementary Table 7) bind ADP and ATPγS, and all mutations except F360A/P reduce the ATPase activity. The NTD position (‘up’ versus ‘down’) is linked to nucleotide state (apo/ATP versus ADP) with the exception of D304N (Supplementary Fig. 7) and F360P (Supplementary Fig. 8), which assume the ‘down’ conformation in the presence of slowly hydrolysable ATP analogues. The ‘up’ conformation of the apo state is not compromised in any mutant. Before ATP hydrolysis, the side chain of D304 hydrogen-bonds with water molecules coordinating the Mg^2+^ ion. Removing its charge leads to loss of Mg^2+^ and concomitant failure to recognize bound ATP and assume the ‘up’ conformation.

**Extended Data Fig. 6:**
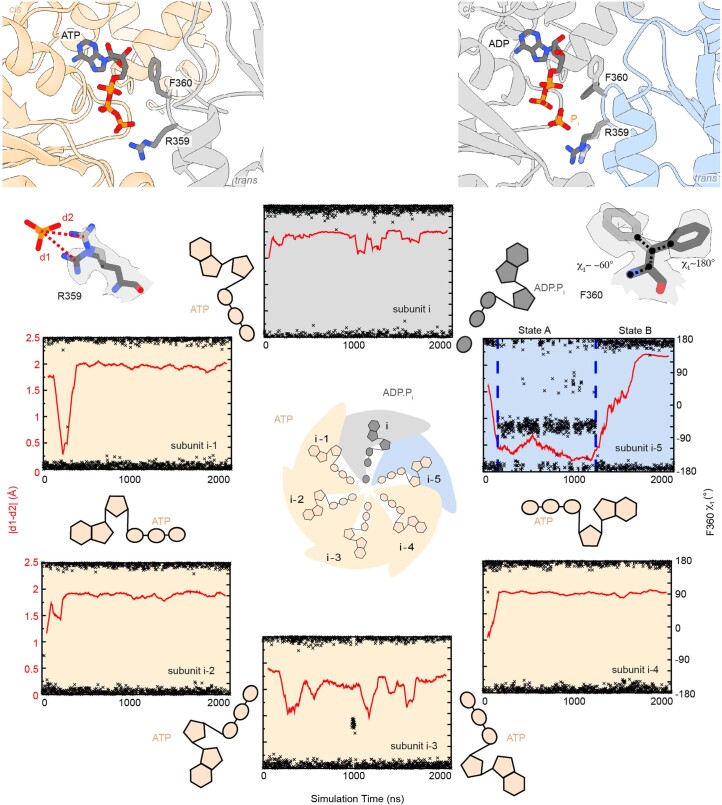
Comparison of active sites in ATP *vs*. ADP·P_i_ states in MD simulations. The structural and dynamical features of arginine finger residues R359 and F360 at the D1 active site were evaluated over the 2 µs MD trajectory of a p97 hexamer with five active sites occupied with ATP and one with ATP cleaved *in silico* into ADP and P_i_. Left ordinate: the distances d1 and d2 between R359-N^η1^/N^η2^ and the P atom of the P_i_ ion reflect the side chain conformation of R359; right ordinate: χ_1_ angle of F360. While all ATP-bound subunits show a stable topology throughout, the ADP·P_i_-bound subunit undergoes a transition when the P_i_ ion moves between states A and B, coupled to a flip of the F360 side chain. On top, MD snapshots from the respective active sites are shown highlighting R359 and F360. Source data

In the ADP·P_i_ state, F360 equally populates two rotamers, while the static ATPγS state shows a preferential F360 *χ*_1_ dihedral of 180° (refs. ^5,28^). This is echoed in the MD simulations, where it is only upon hydrolysis that F360 is unlocked and transiently dissociates from the helix α_407–423_ (Extended Data Fig. 6). This ability of F360 to pull the arginine finger loop towards helix α_407–423_ could be essential to maintain the NTD in the ‘up’ state. The critical role of F360 is underpinned by its conservation in p97 homologues but absence in AAA+ proteins without an NTD (Fig. 3a and Supplementary Fig. 5). Disease-associated p97 mutants lack this rotamer switch^29^, display a dynamic NTD^30^ and no long-lived ADP·P_i_ state^11^. F360 is the only site where mutation entails a gain of ATPase function. Mobility at this site is indeed linked to ATP processing: crosslinking C360 to C410 abolishes ATPase activity (Fig. 3d).

### Determinants of ATP-hydrolysis competence

Real-time NMR establishes that mutants with low ATPase activity (P246T, P247A/K, E305Q and R359K; Fig. 3b) still form an ADP·P_i_ state, pointing to slow product release but intact ATP hydrolysis. However, the N348Q mutant with no measurable ATPase activity displays only the NTD ‘up’ state in the presence of ATP (Supplementary Fig. 9). The sensor residue N348 is thought to position the water molecule for nucleophilic attack on ATP^31^. To recapitulate the suppression of ATP hydrolysis, we evaluated the frequency of reactive conformations at the D1 active site in MD simulations of wild type versus N348Q p97 (Fig. 3e). Although three of five ATP-bound subunits sampled reactive conformations with high frequency in the wild type, all but one subunit were practically inactive in the mutant (statistics are provided in Supplementary Fig. 10). The longer side chain of Q348, which congests the active site, disfavours the proper geometry for ATP hydrolysis. E305 is thought to activate a water molecule for attack on the γ-phosphate of bound ATP^15,31^. The E305Q mutation strongly reduces the ATPase activity of D1^29^. However, the rate-limiting step of the catalytic cycle of this mutant remains product release^11^.

### Dynamics in the sensor loop are coupled with product release

**Fig. 4:**
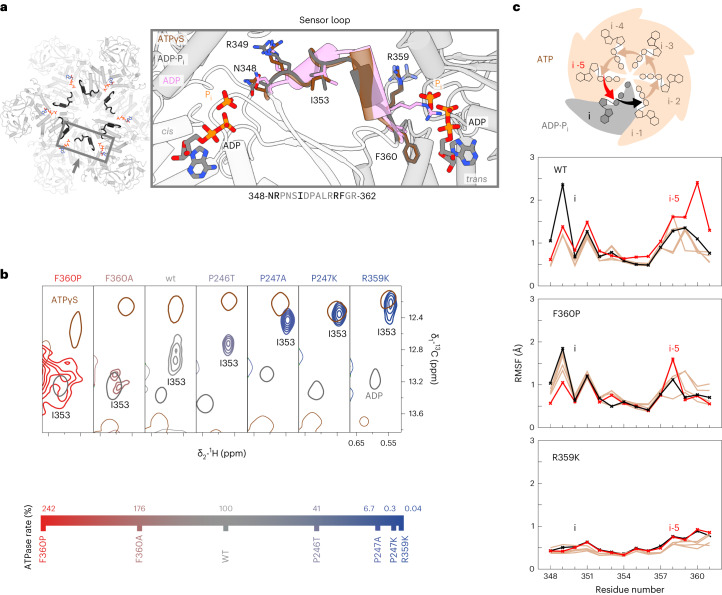
Inter-subunit communication channel connects active sites. **a**, The sensor loop bridging the N348 of one nucleotide binding pocket to F360 of the counterclockwise adjacent pocket changes the conformation between the ATP(γS)- and ADP-bound states. In the ADP state, R349–I353 form a 3_10_ helix, but in the ADP·P_i_ state, this transition is incomplete, making this loop the last structural element to convert after ATP hydrolysis (see the Ramachandran analysis in Extended Data Fig. 7). **b**, The signal of the I353 Cδ_1_-methyl group in the middle of the loop displays line broadening in the ADP·P_i_ state of wild-type p97. For hydrolysis-competent mutants, the ATPase activity correlates with the extent of conversion from an ATPγS-like to an ADP-like conformation. This correlation could reflect the coupling of loop motions to product release, the rate-limiting step of the ATP-hydrolysis cycle. **c**, Cα-RMSF fluctuations quantify the deviation of residues from their average position over the course of the 2-µs MD trajectory. In wild-type p97, mobility is pronounced in sensor loops neighbouring ADP·P_i_-bound but not ATP-bound pockets. In the hyperactive F360P mutant, mobility is increased in all subunits, irrespective of the nucleotide state. In the hypoactive R359K mutant, it is strongly decreased in all subunits. The corresponding RMSD analysis is shown in Supplementary Fig. 11, and excerpts from the MD simulations in Supplementary Videos 2 and 3. Source data

**Extended Data Fig. 7:**
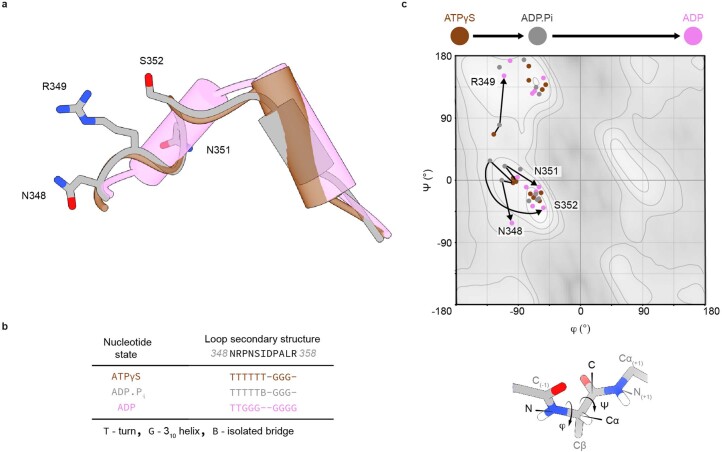
Conformational changes in the sensor loop. **a**, Superposition of the sensor loop in different nucleotide states. The sensor loop extends from the sensor residue N348 of one subunit to the arginine finger of the adjacent subunit. Its secondary structure is more similar between ATPγS (brown) and ADP·P_i_ (grey) states than between ADP·P_i_ and ADP (pink) states, making it the last structural element in D1 to transition from pre-hydrolysis to post-hydrolysis conformation. **b**, For residues 350–352, the STRIDE algorithm^76^ detects a turn for ATPγS and ADP·P_i_ states and a 3_10_ helix for ADP state. Analyses were performed on the following models: ATPγS: 5ftn (ref. ^5^), ADP·P_i_ state A: this work, ADP: 5ftk (ref. ^5^). **c**, The structural transition of the sensor loop is documented by a Ramachandran plot analysis^77^. Residues R359, F360 as well as N348, R349, N351 and S352 undergo dramatic changes in backbone conformation with the progression of ATP-hydrolysis cycle (indicated by the direction of the arrow). Residues 349–352 in the first half of the loop change little between ATPγS to ADP·P_i_ states, but undergo a turn-to-helix conversion between ADP·P_i_ and ADP state. The definitions of backbone dihedral angles φ (C, N, Cα, C) and ψ (N, Cα, C, N) are indicated below.

Sequential ATP hydrolysis around the multimer ring has emerged as a plausible operation mode for AAA+ proteins^10,32^. A communication line between the active sites of adjacent subunits must underlie such coordination. We hypothesized that the ‘sensor loop’ (Fig. 4a) could assume this function in p97 D1. Part of this loop converts from turn to 3_10_-helix between the ATPγS and ADP states. The ADP·P_i_ state, however, still exhibits a conformation similar to ATPγS, unlike the rest of the D1 domain (Extended Data Fig. 7). Transitions of the loop can be monitored via the central reporter residue I353. Its NMR signals are distinct in the ATPγS and ADP states and exchange-broadened in the ADP·P_i_ state (Fig. 4b), indicative of a loop motion occurring on a millisecond timescale. A mutant series reveals a correlation between the extent of turn–helix conversion and the ATP-turnover rate. Globally, all mutants display the spectral signature of the ADP·P_i_ state with NTD in the ‘down’ conformation. The I353 signals of the hyperactive F360P/A mutants are notably broadened, and at the other extreme, the signal of the hypoactive R359K mutant overlaps with the ATPγS state. Apparently, the loop does not respond to ATP hydrolysis with a structural or dynamical change in this mutant.

We compared the residue-wise Cα root-mean-square fluctuation (RMSF) of the sensor loop in the MD simulation of a p97-ND1L hexamer with five ATP and one ADP·P_i_ bound subunits (Fig. 4c). For the wild type, ATP hydrolysis increases structural fluctuations—the two subunits lining the ADP·P_i_ active site display distinct profiles with increased mobility. In simulations of mutant p97, however, loop mobility is increased for the hyperactive F360P and decreased for the hypoactive R359K mutant, irrespective of the nucleotide state. The RMSF of R349 in the wild type substantially increases when the adjacent active site is in the ADP·P_i_ state. The cryo-EM densities of R349 in the ADP·P_i_ map are not well defined, suggesting residual flexibility.

In summary, NMR and MD analyses concur that mobility and the propensity for 3_10_-helix formation in the sensor loop are linked to the ability of product release. As the loop directly connects adjacent active sites, it is conceivable that its structural transition triggers sequential ATP hydrolysis events, which have been observed when p97 is working asymmetrically in the presence of cofactors and substrates^8^.

### Energetics of phosphate binding and release

**Extended Data Fig. 8:**
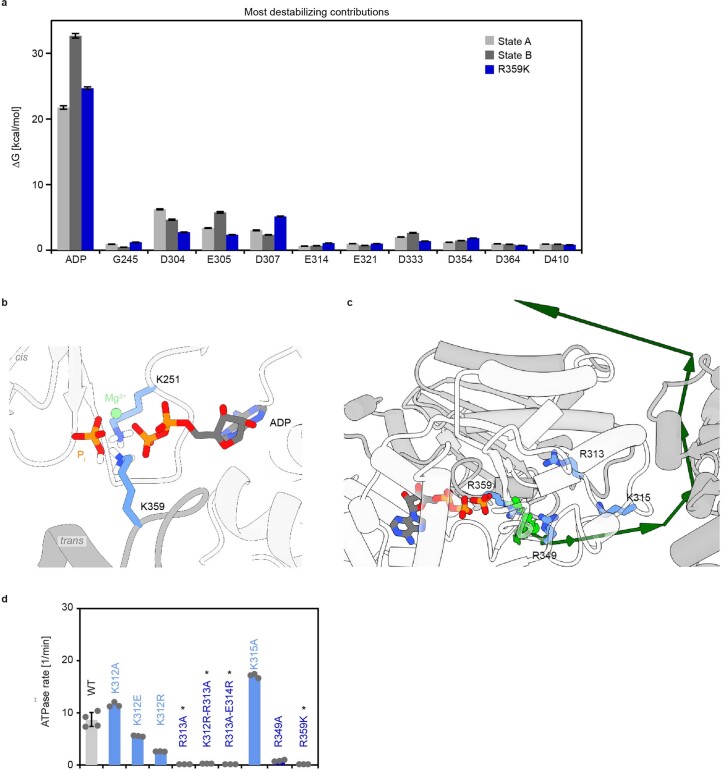
Contributions of active site residues to P_i_ destabilization and release. **a**, Energy decomposition of MMPBSA calculations identifies residues that destabilize the ADP·P_i_ states A and B the most. Especially state B is destabilized by the repulsion between ADP and P_i_. The overall binding free energy estimated by MMPBSA calculations considers non-bonded interactions (Coulombic, van-der-Waals) between protein and ligand as well as changes in solvation free energy between unbound and bound states. In p97, the most important factors are polar solvation energies (destabilizing) and electrostatics (stabilizing). Only the electrostatic contributions differ substantially between the calculations for states A, B and R359K mutant. Data are presented as mean values. Error bars represent s.e. Statistics are derived from n=250 conformations extracted from a single MD simulation. **b**, Lysine side chains contributed by K251 and K359 symmetrically stabilize the ADP·P_i_ state of the P_i_-release deficient mutant R359K. **c**, Side view of the P_i_ dissociation trajectory (top view in Fig. 5b) shows the P_i_ ion leaving p97 through the central pore via the top of the hexamer. **d**, ATPase activities of p97-ND1L with mutations of arginine and lysine residues that contact the P_i_ ion during dissociation (Fig. 5d). Note that the removal of arginines but not lysines strongly reduces ATP turnover and that the mutation R→K does not sustain ATPase activity. The ATPase activity also cannot be recovered by introducing R at neighbouring sites (as in K312R-R313A or R313A-E314R). Data are presented as mean values. Error bars represent s.d. for n = 4 biologically independent replicates. ATPase rates were determined in n=3-4 replicates as indicated by corresponding data points. Source data

The ADP·P_i_ state of the R359K mutant is particularly long-lived, suggesting that a specific interaction mode of the P_i_ ion with R359 might be a prerequisite to induce product dissociation. In MD simulations, its guanidinium moiety interacts exclusively with the P_i_ ion and not with ADP. By contrast, the side chain of K359 preferentially coordinates between the P_i_ and the β-P of ADP, where it shields negative charges and stabilizes the ADP·P_i_ complex in a similar manner as the Mg^2+^ ion (Extended Data Fig. 8b and Supplementary Video 2). Although the K359 mutant populates only state A, the wild-type features transitions between states A and B.

**Fig. 5:**
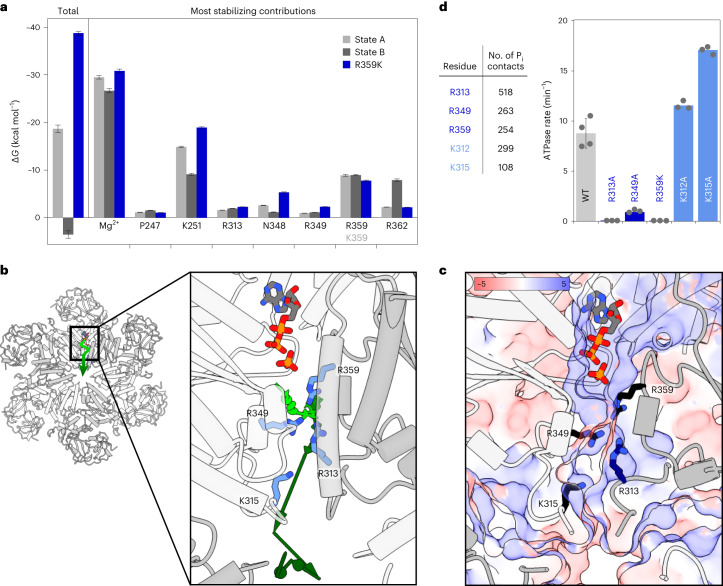
Pathway of phosphate dissociation. **a**, Free energies of ADP·P_i_ complexes from MMPBSA^33^ calculations. Energy decomposition identifies entities that stabilize the leaving P_i_ ion most strongly: the Mg^2+^ ion and the side chain of K251. Electrostatic terms but not solvation terms are responsible for energy differences between the states. Data are presented as mean values. Error bars represent s.e. Statistics are derived from *n* = 250 conformations extracted from a single MD simulation. **b**, Illustration of phosphate dissociation (arrows, light to dark green) from the active site towards the central pore derived from an MD trajectory (a side view is shown in Extended Data Fig. 8c). **c**, Same view as **b**, colour-coded according to electrostatic potential in units of kT e^−1^ (APBS ref. ^42^), defining a positively lined channel. **d**, Left: positively charged residues in D1 with the highest number of contacts to the dissociating P_i_ (defined by a distance below 3 Å in a single frame) in the MD trajectory. Right: ATPase activities of the corresponding p97-ND1L point mutants. The mutation of arginine but not lysine residues causes a drastic decrease in ATPase activity (additional mutants are shown in Extended Data Fig. 8d). Data are presented as mean values. Error bars represent s.d. for *n* = 4 biologically independent replicates. ATPase rates were determined in *n* = 3 or 4 replicates, as indicated by the corresponding data points. Source data

To quantify the stability of wild-type states A versus B versus K359 mutant, we conducted MMPBSA (molecular mechanics Poisson–Boltzmann surface area^33^) calculations to estimate the interaction energy of the P_i_ ion to the ADP·P_i_ state of p97. This method also allows the decomposition of total free energies into the most stabilizing (Mg^2+^, K251; Fig. 5a) and destabilizing (ADP; Extended Data Fig. 8a) contributions. We here consider the P_i_ ion as the ligand and p97–ADP–Mg^2+^ as the receptor. Although R359 interacts with the P_i_ ion both in the simulations and cryo-EM, it is not necessary for achieving a stable ADP·P_i_ state—the presence of a Mg^2+^ ion bridging ADP and P_i_ is sufficient. In contrast to the stable binding pose in state A of the wild type (Δ*G* ≈ −19 kcal mol^−1^) and K359 mutant (Δ*G* ≈ −39 kcal mol^−1^), P_i_ binding is predicted to be unstable in state B (Δ*G* ≈ +3.5 kcal mol^−1^), chiefly due to the repulsion between ADP and P_i_. Thus, transitions from state A to B could mark the onset of P_i_ dissociation events in wild-type p97. By contrast, the R359K mutant has no state B equivalent with positive Δ*G* and an even stronger stabilization of the P_i_ ion compared to wild-type state A. This combination manifests in inefficient product release and low ATP turnover rates.

Over the 2-µs simulation, the ADP·P_i_ state remains stable as long as cations bridging ADP to P_i_ are present. We therefore expedited complex dissociation by removing the Mg^2+^ ion artificially. In the resulting trajectory (Fig. 5b), the P_i_ travels from the active site towards the centre of the hexamer to dissociate through the central pore along a channel lined by positive charges (Fig. 5c). Intriguingly, the ion is handed over from R359 of the *trans*-acting subunit to R349 of the *cis*-acting subunit, which mark the end and the start of the respective sensor loops.

To probe the effective contributions of individual residues to P_i_ evacuation, we generated a series of point mutants altering charges or exchanging Arg ↔ Lys and determined their ATPase activities (Fig. 5d and Extended Data Fig. 8d). Removal of Arg but not Lys residues strongly reduces the ATPase rates; the replacement of lysine by arginine cannot rescue slow-release mutants. In line with the MMPBSA analysis, the sequential interaction of the P_i_ ion with R359, R349 and R313 could mark the onset of P_i_ release. The active site and dissociation channel are finely evolved to ensure both the initial stabilization and eventual evacuation of the reaction products.

### Signalling of hydrolysis-induced domain motion

**Fig. 6:**
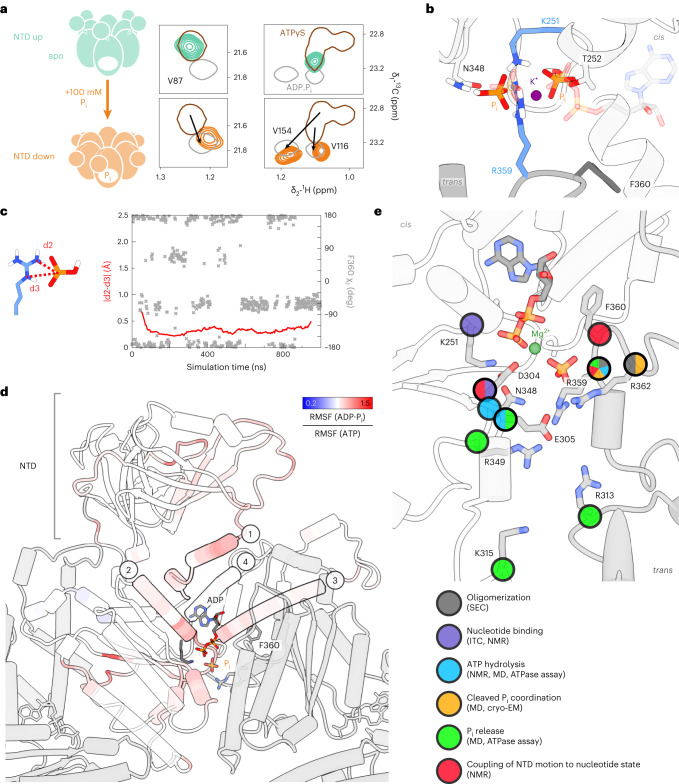
Allosteric control of NTD motion. **a**, NMR probes indicate a conformational change of the NTD induced by the addition of 100 mM P_i_ ions to apo p97, reflected by peak shifts from ‘up’ (ATPγS-like) to ‘down’ (ADP·P_i_-like) position (the titration is shown in Extended Data Fig. 9a). This P_i_ concentration is above physiological intracellular levels. The same effect was observed for arsenate and sulfate ions (Extended Data Fig. 9b). **b**, Snapshot from MD simulations (Supplementary Video 4) of the apo D1 nucleotide binding pocket. Two P_i_ ions mimic the ADP·P_i_ state (transparent) and occupy the same positions as the β-P of ADP and the leaving P_i_, bridged by a K^+^ or Na^+^ ion from the solvent. No Mg^2+^ ion is necessary to stabilize this arrangement in simulation or experiment. **c**, The MD trajectory of P_i_-bound p97 reveals a mobile F360 side chain that switches between rotamers corresponding to states A and B (Fig. 2b); R359 stably coordinates a P_i_ ion via its N_ε_ atom and one amino group. **d**, Top view of one D1 subunit colour-coded according to the ratio of the backbone RMSF of ADP·P_i_ state B over the ATP state, each sampled over 800 ns. Red colour indicates an increase in mobility upon hydrolysis, observed in (1) helix α_191–199_ and the NTD-D1 linker, (2) helix α_251–262_ extending from the Walker A motif to the NTD-D1 interface, (3) helix α_407–423_ to which F360 associates transiently and (4) helix α_374–387_ running past the adenine moiety of the nucleotide (increased mobility in state A only, cf. Extended Data Fig. 10a). **e**, Summary of the function of residues at the p97 D1 active site. Categories were assigned either according to structural contributions evident from MD or cryo-EM or according to mutagenesis-induced defects in ATP processing. ITC, isothermal titration calorimetry. Source data

The NTD position is controlled allosterically by the nucleotide state in D1. In the apo and ATP(γS)-bound states, it is detached from and elevated above the D1 domain. In the ADP·P_i_ and ADP-bound states, it moves downward to form an extensive interface. This process is not observed in our MD simulation, which captures only 2 µs immediately after hydrolysis. However, we could identify the minimal structural signal to stabilize the NTD to the coplanar position by titrating P_i_ ions to apo p97-ND1L (Fig. 6a). Simulations corroborate the experimental result, suggesting that two P_i_ ions bridged by a monovalent cation bind stably to apo p97. They adopt the same positions as P_i_ and β-P in the ADP·P_i_ state (Fig. 6b). The resulting complex reproduces the two-pronged interaction between P_i_ and the R359 guanidinium group, as well as the rotamer switches of F360 *χ*_1_ (Fig. 6c) observed in ADP·P_i_ state A.

**Extended Data Fig. 10:**
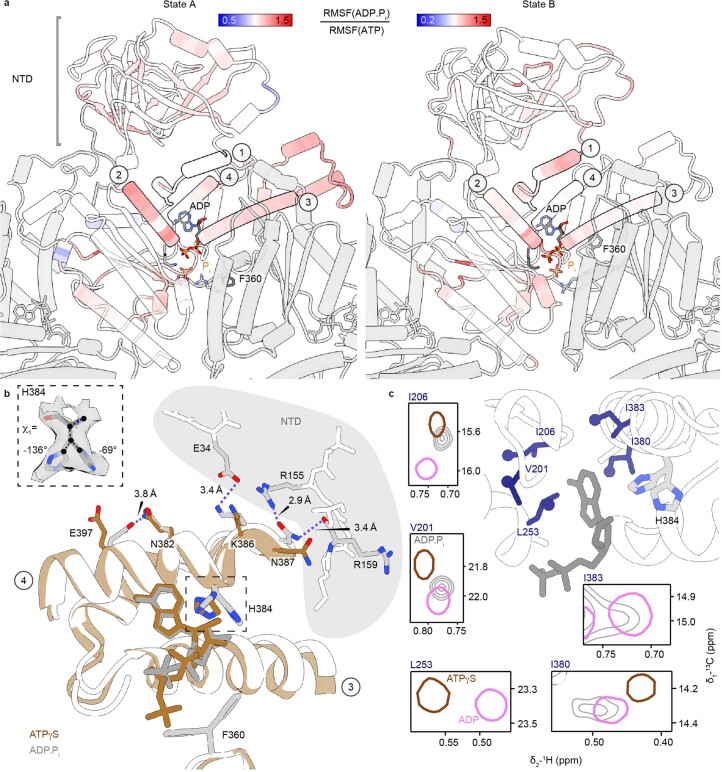
Effects of ATP hydrolysis on protein mobility and structure. **a**, The RMSF of Cα atoms over the 2 µs MD trajectory (same as Extended Data Fig. 6) quantifies protein mobility at the level of individual residues. The ratio of the RMSF values of the ADP·P_i_-bound subunits in states A (left) and B (right) over the average of the five ATP-bound subunits is shown as a heat map on the respective MD snapshots. Increased mobility upon hydrolysis (red shades) is observed propagating from the active site. State B is overall less mobile than state A. Regions of interest include: (1) helix α_191-199_ and the NTD-D1 linker; (2) helix α_251-262_ extending from the Walker A motif to the NTD-D1 interface; (3) helix α_407-423_ to which F360 associates transiently in *trans*; (4) helix α_374-387_ running past the nucleotide towards the NTD-D1 interface displays increased mobility in state A only. **b**, Two side chain rotamers of H384 are uniquely observed for the ADP·P_i_ state. They belong to a larger interaction network between D1 and NTD, which enables the conformational change that locks the NTD into the ‘down’ position. In comparison to the ATPγS structure, the ADP position turns 7° in the ADP·P_i_ structure, enabling an interaction between the ribose moiety and H384. The C-terminus of helix α_374-387_ forms an interface with the NTD (stick representation) only in the ‘down’ state, where N387 forms a tight network with residues R155 and R159. Meanwhile, N387 flips away from this interface when the NTD is in the ′up′ position (*c.f*. Supplementary Video 5). The insert shows the experimental cryo-EM density for the side chain rotamers of H384 at a threshold level of 0.0045. Helices (3) and (4) as defined in panel a are labelled. **c**, The NMR signals of nearby residues are sensitive both to ATP hydrolysis (ATPγS *vs*. ADP·P_i_) and to P_i_ release (ADP·P_i_ *vs*. ADP). The location of the methyl probes is visualized on the ADP·P_i_ state structure.

From the active site, structural changes induced by ATP hydrolysis must be relayed towards the NTD, where they ultimately induce a downward motion. We evaluated the RMSF of Cα atoms over the trajectory and visualized their ratio between the ADP·P_i_ and ATP subunits as a heatmap on the MD structure (Fig. 6d and Extended Data Fig. 10a). The interaction between the leaving P_i_ and R359 induces the dissociation (state A) and re-association (state B) of F360 with respect to helix α_407–423_ and thereby increases the plasticity of the arginine finger loop and the entire active site. This increased plasticity propagates towards the periphery of D1: first along the NTD-D1 linker; second along the helix extending from the Walker A motif towards the NTD; third along helix α_407–423_ from the ribose moiety towards the *trans*-acting subunit; and fourth from the adenine moiety along helix α_374–387_ towards the NTD. The latter effect is reflected in the cryo-EM map: only in the ADP·P_i_ state does H384 display a second side-chain rotamer that contacts the ribose. The nucleotide is thus slightly repositioned, helix α_374–387_ shifts with respect to the ATPγS state, leading to a flipping of N387 located at the end of the helix_._ The repositioning of N387 in turn enables the formation of an electrostatic network with NTD residues that fix the ‘down’ state (Extended Data Fig. 10b and Supplementary Video 5). Even though P_i_ ions are sufficient to evoke the ‘down’ state of the NTD, the nucleoside moiety still contributes to the signalling of ATP hydrolysis. The MD trajectory cannot capture the downward motion of the NTD, yet it pinpoints early dynamical changes that could eventually pave the way for this large-scale conformational transition.

## Discussion

Resolving the mechanism by which ATP hydrolysis is catalysed and the concomitant release of chemical energy is conveyed to mechanical motion is a major challenge in the field of enzymology. Structures of transiently captured intermediates allow dissection of the catalytic cycle into experimentally grounded snapshots. The ADP·P_i_ intermediate, in which the bond between γ- and β-phosphate groups has been cleaved but neither the P_i_ ion nor ADP has been released yet, has been poorly characterized. Its existence was first postulated for myosin^12^, in which a stable ADP·P_i_ complex can be artificially induced with exogenous P_i_—a property shared by myosin^24^, F-actin^23^ and Hsc70^22^. However, structures of such stable complexes may not reflect the authentic short-lived states during enzymatic hydrolysis, nor do they cover any members of the most prevalent nucleotide-binding fold, the P-loop NTPases^34^.

We report here the 2.6-Å cryo-EM structure of the human ATPase p97 captured in a transient ADP·P_i_ state, which converges with MD simulations of the same state. Mutagenesis and NMR analyses identify the contributions of active-site residues to ATP turnover, as summed up in Fig. 6e. The structures capture molecular motions that accompany ATP hydrolysis, where the cleaved P_i_ travels together with the Mg^2+^ ion as a contact ion pair. In the metastable ADP·P_i_ state, the Mg^2+^ ion is held in place by Walker B residue D304 and by the β-P of ADP. The release of P_i_ is coupled with rotamer exchanges in the arginine finger loop. A further rotamer exchange of H384 triggers a conformational transition that could ultimately direct the large-scale motion of the NTD. A stable Mg^2+^·P_i_ complex and water networks have been observed for F-actin^35,36^ and Hsc70^37,38^, indicating a common mechanism whereby the Mg^2+^ ion plays a key role in P_i_ release. Indeed, the ADP·P_i_ state remains stable as long as bridging cations are present in simulations. Notably, in a GHL ATPase, the switch of a lysine residue near the nucleotide was proposed to trigger P_i_ release, which parallels our finding of an arginine finger rotamer switch^39^.

We have identified a loop connecting the sensor I motif to the arginine finger of the counterclockwise subunit. Its ability to transition from turn to 3_10_-helix between ATP, ADP·P_i_ and ADP states is correlated to efficient product release. p97 is a prototype member of the AAA+ superfamily, and ADP·P_i_ states are frequently invoked in mechanistic models of these ring-shaped oligomers^14,40,41^. A consensus has emerged that ATP hydrolysis proceeds counterclockwise in substrate-engaged AAA+ proteins^10,32^. ADP release disrupts the subunit interface and causes the respective subunit to move to the bottom of the spiral staircase and disengage from the substrate^40,41^. Such models presume efficient inter-subunit communication elements, such as the sensor loop identified in p97.

A transient ADP·P_i_ state for p97 has now been captured. A high kinetic barrier to P_i_ dissociation, rendering release rate-limiting, could be a speciality of p97 D1—the conserved phenylalanine residue in the arginine finger of p97 D1 is replaced by proline in p97-D2 and many AAA+. We show that F360 rotamer states regulate the NTD position and are coupled with P_i_ release. Although ATP turnover in D1 is linked to NTD motion, D2 drives substrate translocation^8,9^.

Our methodology delineates a general strategy to overcome resolution limits in the characterization of short-lived and heterogeneous enzymatic reaction intermediates. Single-particle cryo-EM affords the bulk of structure determination, MD simulations validate the interpretation of the map at the critical active site and introduce a time axis to connect multiple structural states, and NMR assesses dynamical changes coupled with enzymatic events.

## Methods

All chemicals were purchased from Carl Roth or Sigma-Aldrich, unless otherwise stated.

### Production of recombinant p97 protein

For the NMR experiments, full-length human p97 (UniProt P55072) and p97-ND1L (residues 1–480) were produced with N-terminal His_6_-tag and tobacco etch virus (TEV) cleavage site as previously described^11,30^. Point mutations were introduced using site-directed mutagenesis (New England Biolabs). The following mutants were generated: for p97-ND1L—P246T, P247A, P247K, K251A, D304N, E305Q, K312A, K312E, K312R, K312R-R313A, R313A, R313A-E314R, K315A, N348Q, R349A, R359A, R359A-R362A, R359K, F360A, ΔCys-F360C-A413C (ΔCys: C69V-C77V-C105A-C174A-C184W-C209V-C415A), F360P and R362A; for full-length p97—E305Q-E578Q mutant for ssNMR experiments. For cryo-EM experiments, glutathione-*S*-transferase (GST)tagged p97 was cloned into a pGEX6p1 vector.

All proteins were over-expressed in *Escherichia coli* BL21(DE3) cells. For solution-state NMR, perdeutration and selective labelling with I-δ_1_-[^13^CH_3_], V/L-γ_1_/δ_1_(*proR*)-[^13^CH_3_,^12^CD_3_] and M-ε_1_-[^13^CH_3_] were achieved as previously described^11^. Cells were induced with 0.5–1 mM isopropyl β-d-1-thiogalactopyranoside 1 h after the addition of the selective labels, and grown overnight at 16–18 °C.

His_6_-tagged p97 constructs were purified^30^ using Ni^2+^-NTA affinity chromatography followed by TEV protease cleavage, followed by size-exclusion chromatography (SEC) on a Superdex 200 column (Cytiva). Bound nucleotide was removed via apyrase digestion (New England Biolabs) in the presence of 2 mM dithiothreitol (DTT) and 4 mM CaCl_2_, overnight at room temperature, followed by another run on a Superdex 200 column. Protein concentrations were determined photometrically.

GST-tagged full-length p97 was bound to GST Sepharose beads (Cytiva). After washing (PBS pH 7.4, 1 mM DTT), p97 was eluted (50 mM Tris pH 8.0, 10 mM glutathione) and subjected to GST tag cleavage by HRV3C protease^43^. The protein was then applied to a Resource Q column (Cytiva) and eluted with a NaCl gradient (50 mM Tris pH 8.0, 0–1 M NaCl), followed by further purification using a Superose 6 Increase column (Cytiva) in 25 mM Tris pH 8.0, 150 mM NaCl, 1 mM MgCl_2_ and 0.5 mM tris(2-carboxyethyl)phosphine (TCEP). Finally, the sample was buffer-exchanged to storage buffer (50 mM HEPES, pH 8.0, 150 mM NaCl, 1 mM MgCl_2_, 0.5 mM TCEP) before snap-freezing.

### NMR sample preparation

For solution-state NMR experiments, samples of perdeuterated p97 labelled with *proR*-^13^CH_3_-ILVM were buffer-exchanged (25 mM HEPES pH 7.5, 25 mM NaCl, 5 mM TCEP, 100% D_2_O) to concentrations in the range of 50–200 μM. For assessment of the different nucleotide states, the protein samples were supplemented with 5 mM ADP or 4 mM MgCl_2_ and 5 mM ATPγS or AMP-PNP (Jena Bioscience). The set-up of the ATP regeneration system was achieved as previously described^11^. For solid-state NMR measurements, 3 mg of ND1L-E305Q or fl-E305Q-E578Q at natural isotopic abundance was dialysed (25 mM HEPES pH 7.0, 50 mM NaCl, 5 mM TCEP, 100% H_2_O), supplied with the regeneration system and sedimented into 1.3-mm magic-angle-spinning (MAS) rotors (Bruker) using filling tools (Giotto Biotech).

### NMR titrations

The apo state of wild-type p97-ND1L was titrated with P_i_ in several steps from 0 up to a final concentration of 100 mM from an 800 mM Na_2_HPO_4_ pH 7.5 stock solution. All mutants were supplied with P_i_ to a concentration of 100 mM in one step.

Mimics of P_i_ ions were added to the apo state of wild-type p97-ND1L in one step to a final concentration of 100 mM (stocks: Na_2_HAsO_4_ dissolved to 300 mM; Na_2_SO_4_ dissolved to 1 M; stocks adjusted to pH 7.5).

### NMR spectroscopy

Solution-state NMR experiments were conducted on Avance III Bruker spectrometers equipped with TCI cryo probes at field strengths corresponding to proton resonance frequencies of 800, 900 and 950 MHz. Sample temperatures during data acquisition were 37 °C (all apo states), 40 °C (all K251A spectra; F360A/P spectra in the presence of ATP) or 50 °C (all others).

Solid-state NMR experiments were performed on an Avance III 800 MHz Bruker spectrometer under 45 kHz MAS at 5 °C (ref. ^11^). Cross-polarization-based experiments were measured interleaved with directly pulsed experiments for reaction control. Chemical shifts were referenced to internal sodium trimethylsilylpropanesulfonate (DSS).

The experimental parameters are listed in Supplementary Tables 1 and 2. All spectra were processed using TopSpin (Bruker; v 3.5 and 3.7) and analysed using CcpNmr Analysis (CCPN, v 2.5.2)^44^. The ^31^P spectrum (Fig. 1b) was fitted using Mnova 11.0 (Mestrelab).

### Cryo-EM

#### Grid plunging and cryo-EM data acquisition

Purified p97 was concentrated to approximately 4 mg ml^−1^ and incubated in the ATP regeneration system (4 mM ribose-5-phosphate, 4 mM MgCl_2_, 50 mM KCl, 13.3 U pyruvate kinase, 50 mM phospho-enol pyruvate, 10 mM ATP) for 20 min at 37 °C. Octyl-beta-glucoside at a concentration of 0.05% was added just before plunge-freezing. A 3-μl sample was blotted on glow-discharged Quantifoil Cu R2/1, 200 mesh grids. Plunge-freezing was performed with a Vitrobot Mark IV (Thermo Fisher Scientific) in a chamber equilibrated at 10 °C with 100% humidity. Images were acquired with a Titan Krios G4 (Thermo Fisher Scientific), with a Falcon 4 detector (Thermo Fisher Scientific) mounted after a Selectris energy filter with slit width at 15 eV. A total of 10,011 images were collected using EPU (v 3.1) with aberration-free image shift (AFIS), at ×165,000 magnification (0.72 Å pix^−1^). Each image had an exposure of 40 e Å^−2^, with an exposure rate of 5.41 e pix^−1^ s^−1^. The nominal defocus range was from −0.9 to −2.2 μm.

#### Cryo-EM image processing

Each image consisted of 931 electron-event representation (EER) frames. Initial drift correction was performed with Motioncorr^45^ as implemented within Relion^46^, such that a grouping of 23 EER frames was used. Contrast transfer function (CTF) parameters were estimated by CTFFIND4^47^. A total of 693,686 particles including both single and double hexamers were picked with Cryolo^48^ using a p97-trained network. Relion 4.0^49^ was used for subsequent data-processing. All particles were first extracted in bin2 and subjected to initial cleaning up by 2D and 3D classification applying *C*1 symmetry. Owing to the higher resolution achieved by double-ring particles in comparison to single-ring particles, 199,453 double-ring p97 particles but not the single-ring particles were selected for further processing. The particles were re-extracted without binning in a box of 330 pixels, and 3D refinement reached a global resolution of 2.98 Å after CTF, magnification and higher optical aberration corrections. To obtain a high-resolution map for in-depth analysis of the D1–D2 domain, Bayesian polishing was performed and followed by subtraction of single-ring from double-ring particles, which effectively doubled the total number of particles to 398,906 and led to a reconstruction of 2.83 Å with *C*6 symmetry applied. A subset of 181,651 particles were identified by a focused 3D classification without alignment, which produced a 2.64 Å map (*C*6 symmetry applied) after CTF and higher optical aberration correction. As a final push of resolution, a limit to use only particles with closer defocus than −1.7 μm reduced the number of particles to 86,760, yielding a map of 2.61 Å (*C*6 symmetry applied).

Because the NTD domain is typically more flexible than the D1–D2 ring, the following image processing was performed to improve the map quality of the NTD domain. The 181,651 computationally subtracted single rings were reverted back to double rings, and duplicated particles were removed such that 125,454 particles remained. Subtraction of single-ring from double-ring particles yielded a total of 250,908 particles. After further 3D refinements and 3D classification without alignment, 112,231 particles were selected and a signal subtraction was performed to focus on only one p97 subunit, which finally yielded a map of 3.27 Å with sufficient NTD density quality for interpretation. Acquisition parameters and statistics are listed in Supplementary Table 3.

#### Initial cryo-EM model building

The published models PDB 5FTM and 5FTL ref. ^5^ were used as a starting point for model building. The model was first rigid-body-docked to the D1–D2 ring focused map and NTD focused map, followed by manual adjustment in Coot^50^. The model was then refined by phenix.real_space_refine. A composite whole map of p97 was constructed by combining the two focused refined maps of the D1–D2 ring and NTD using phenix.combine_focus_maps^51^. The models were docked into the composite maps to generate a complete model of p97 (Supplementary Table 4). The model was then subjected to MD simulation for analysis of the P_i_ and Mg^2+^ ion positions.

#### MD simulations

The MD simulations were performed using the graphics processing unit accelerated version of pmemd^52^, as distributed with the AMBER18^53^ package. For proteins, the ff14Sb^54^ force field was used, whereas water molecules were described with the SPC/E^55^ model. To model the crucial Mg^2+^ ions as accurately as possible, the 12-6-4LJ model^56^ for divalent ions was used. ATP and ADP parameters^57^ were taken from the parameter database of the University of Manchester. Single and double protonated P_i_ ions (HPO_4_^2−^ and H_2_PO_4_^−^) were parameterized utilizing the GAFF2^58^ force field for organic molecules. For this, RESP^59^ partial charges (Hartree-Fock at 6-31-G* level) were calculated from a structure that was optimized to a gas-phase energy minimum at the B3LYP/TZVP-level. All quantum mechanics calculations were conducted with GAUSSIAN09^60^.

All simulations were performed at 303.15 K and a pressure of 1 atm using the Langevin^61^ thermostat (with a collision frequency of 1 ps^−1^) and the Monte Carlo barostat^62^ (*τ*_p_ = 1.0 ps), respectively. Non-bonded interactions were calculated explicitly until a distance cutoff of 9 Å. Long-range Coulomb interactions were accounted for by the particle mesh Ewald method^63^, and long-range van der Waals effects were described by a dispersion correction model. During the sampling phase, time steps of 4.0 fs were used, enabled by constraining all bonds involving hydrogen atoms^64^ to their equilibrium lengths as well as applying the hydrogen mass repartitioning method^65^. Data analysis was performed using VMD 1.9.3^66^ and CPPTRAJ^67^.

#### Simulation set-up

The MD simulations of wild-type p97-ND1L in the ADP·P_i_ state and point mutations thereof (N348Q, R359K, F360P) were started from the crystal structure with PDB 4KO8 (ref. ^29^), which lacks the D2 subunit. Hexamers were generated from the asymmetric unit, which contains a dimer encompassing residues 14−469. ATPγS was transformed into ATP (in five of six subunits), while ADP and either H_2_PO_4_^−^ or HPO_4_^2−^ was placed in one subunit. The starting position of the in silico-created P_i_ ion was chosen so that it coincided with the position of the former γ-phosphate.

Protonation states of titratable groups were assigned using the PDB2PQR server (https://server.poissonboltzmann.org/pdb2pqr)^42^ at pH 7.4. Only the protonation state of K251 was set manually (to charged). To determine the likely protonation state of K251, two separate simulations were initially conducted of the D1 subunit in the ATP state (starting from the crystal structure with PDB 4KO8 as described above). In these simulations, the charge of K251 has a very large impact on interactions with the nucleotide: neutral K251 does not form substantial interactions, whereas positively (+1) charged K251 constantly binds to the γ-phosphate of ATP. Because the published experimentally determined p97 structures show clear and pronounced interactions between K251 and ATP, all simulations for this project were carried out with positively charged K251. Similarly, the lysine side chain in the R359K mutant was assumed to be positively charged.

The simulations were performed using periodic boundary conditions in octahedral simulation boxes containing ~117,000 water molecules as well as 25 mM NaCl and 50 mM KCl.

#### Final model building

The simulation conducted for the refinement of the cryo-EM structure was started from a preliminary cryo-EM structure of full-length p97. All six D1 binding sites contained ADP + HPO_4_^2−^ + Mg^2+^, and all six D2 binding sites contained ATP + Mg^2+^. The solvent consisted of ~106,000 water molecules and Na^+^ counter-ions. Periodic boundary conditions were applied. Unexplained cryo-EM densities around the D1 pocket were explained by the simulation. In the final model, two states (A and B) of the dissociating P_i_ ions with their corresponding Mg^2+^ ions were identified.

The simulation sampling P_i_ ion dissociation was performed under identical conditions except that the Mg^2+^ ions bridging ADP and HPO_4_^2−^ were removed from the system.

An overview of all conducted simulations is provided in Supplementary Table 5.

Before sampling, a seven-step equilibration protocol was applied to all simulation systems (details are provided in Supplementary Table 6).

#### Free-energy calculations

The interaction free energies (Δ*G*) of P_i_ and ND1L-p97 (wild type in states A and B as well as the mutant R359K) were calculated using the MMPBSA single-trajectory method as implemented in the MMPBSA.py^33^ script, which is part of the AMBER18 package^53^. Because the Poisson–Boltzmann (PB) routine of AMBER18 was unable to recognize one of the atom types in the ATP force field, the PB calculations were performed with flags inp=1 and radiopt=0, which resulted in slightly different nonpolar solvation terms compared to the default settings in AMBER18.

The numbers of processed frames for each system were as follows: wild-type state A, 450 frames; wild-type state B, 400 frames; R359K, 500 frames. A salt concentration of 150 mM was chosen. The dielectric constant for the protein was set to 1.0, and water was set to 80.0.

Conformational entropy contributions were neglected because of the high computational cost and generally low accuracy of these methods. Therefore, the resulting Δ*G* values cannot be directly compared to experimental values. However, our analyses compare very similar systems (slightly different conformations of the same protein and a point mutant thereof), so it can be assumed that errors stemming from this treatment cancel each other to a very high degree.

### Biochemical assays

#### Inter-subunit crosslinking

To apo state p97-ND1L-ΔCys-F360C-A413C, 4 mM DTT was added, then the solution was incubated for 2 h at 30 °C. Reducing agent was removed by gel filtration on a Superdex 200 in crosslinking buffer (20 mM HEPES pH 7.2, 250 mM KCl, 5 mM ethylenediaminetetraacetic acid (EDTA)). Fractions eluting as hexamers were pooled and diluted to 20–60 μM. Crosslinking reagent bismaleimidoethane (BMOE; Thermo Fisher Scientific) was supplied in twofold excess from a 20 mM stock in dimethylsulfoxide, and the solution was incubated for 2 h on ice. After quenching with 50 mM DTT (15 min on ice), excess chemicals were removed via another gel filtration run on a Superdex 200 in the gel filtration buffer. Only protein eluting as the hexamer was pooled for further studies. Successful crosslinking was verified by SDS–PAGE (Supplementary Fig. 13).

#### SEC

The oligomerization states of the various p97 mutants were estimated from the elution profile following SEC on a Superdex 200 Increase 10/300 GL gel filtration column (Cytiva; buffer: 50 mM HEPES pH 7.5, 250 mM KCl, 2 mM MgCl_2_). Size calibration was achieved internally using molecular-weight standards (SERVA).

#### NADH-coupled ATPase assay

The ATPase rates of p97 were determined using an NADH-coupled ATPase assay, as the oxidation of NADH is directly coupled to the rate of ATP hydrolysis. Phosphoenolpyruvate (6 mM), NADH (1 mM), pyruvate kinase (1 U/100 μl), lactose dehydrogenase (1 U/100 μl) and purified protein (1–100 μM) were diluted into the ATPase buffer (25 mM HEPES pH 7.5, 25 mM NaCl, 50 mM KCl, 4 mM MgCl_2_, 0.5 mM TCEP) and distributed into a 96-well plate to a final volume of 120 μl. The reaction mixture was equilibrated at either 37 °C or 50 °C for 5 min before the addition of ATP (2 mM). The decrease in absorbance at 340 nm (coupled to NADH oxidation) was monitored with a SpectraMax iD5 plate reader (Molecular Devices) for 60 min. The rate of NADH consumption was then translated into ATPase rates. ATP-hydrolysis rates (ATP min^−1^) were calculated based on at least three experimental replicates.

#### Isothermal titration calorimetry

Isothermal titration calorimetry measurements were conducted on a MicroCal PEAQ-ITC (Malvern Pananalytical) instrument at 25 °C. Protein samples (10–20 μM) were freshly digested with apyrase as described above, and subsequently run over a Superdex 200 column (Cytiva). The lyophilized commercial nucleotides (100–120 μM; Sigma and Jena Bioscience) were dissolved in identical SEC buffer. The experimental parameters included one 0.4-µl injection followed by 19 × 2-μl injections with 120 s of spacing. Data were analysed using MicroCal PEAQ-ITC Analysis software (V 1.21) using the One Set of Sites binding model. *K*_d_ values were calculated based on at least two experimental replicates.

#### Electrostatic potential calculation

Electrostatic potential calculations were performed using the Adaptive Poisson–Boltzmann Solver (APBS)^42^ with input generated from the cryo-EM derived structure (state A) with amendments from the PDB2PQR program^68^.

#### Sequence alignment

All multiple sequence alignments were done using Clustal Omega^69^. Sequences of AAA+ ATPases having two tandem ATPase domains such as NSF and p97 were edited in Jalview^70^ before domain alignment.

The accession IDs of the sequences used for AAA+ ATPases alignment (Fig. 3a) were P35998 (PSMC2_ *H.sapiens*), P46459 (NSF_ *H.sapiens*), Q16740 (CLPP_ *H.sapiens*), P36776 (LONP1_ *H.sapiens*), Q13608 (PEX6_ *H.sapiens*), Q9Y265 (RUVBL1_ *H.sapiens*), P0ABH9 (ClpA_*E.coli*), P31539 (Hsp104_*S.cerevisiae*) and O43933 (PEX1_*H.sapiens*).

The accession IDs of the sequences used for alignment of p97 from different species (Supplementary Fig. 5) were Q7KN62 (TER94_*D. melanogaster*), Q9P3A7 (CDC48_*S. pombe*), P25694 (CDC48_ *S. cerevisiae*) P46462 (VCP_ *R. norvegicus*), Q3ZBT1 (VCP_ *B. taurus*), P54812 (CDC48.2_*C. elegans*), Q01853 (VCP_*M. musculus*) and P55072 (VCP_*H. sapiens*).

#### Ramachandran plot analysis

RamachanDraw (https://github.com/alxdrcirilo/RamachanDraw) was used to create Ramachandran plots. Torsion angles of residues 348–360 in the cryo-EM structure of the ADP·P_i_ state (this work), the ATPγS state (PDB 5FTN)^5^ and the ADP state (PDB 5FTK)^5^ were considered in this analysis.

#### Visualization

Molecular graphics and analyses were performed with UCSF Chimera 1.16^71^ and ChimeraX 1.4^72^. The violin plot was prepared using seaborn^73^.

### Reporting summary

Further information on research design is available in the Nature Portfolio Reporting Summary linked to this Article.

## Online content

Any methods, additional references, Nature Portfolio reporting summaries, source data, extended data, supplementary information, acknowledgements, peer review information; details of author contributions and competing interests; and statements of data and code availability are available at 10.1038/s41557-024-01440-0.

### Supplementary information

Supplementary Information Supplementary information, including references, Figs. 1–13 and Tables 1–8.

Reporting Summary

Supplementary Video 1 Close-up of the D1 active site from an MD simulation sampled immediately after in silico-transformation of ATP into ADP and P_i_ for 2 μs. A concerted rotamer flip of residues R359 and F360 (bottom) is observed after ~1.2 μs, marking the transition from ADP·P_i_ state A to state B. This switch is accompanied by a shift in the position of the cleaved P_i_ ion and of the Mg^2+^ ion (pink), which stably bridges the P_i_ and ADP (top).

Supplementary Video 2 Close-up of the D1 active site bearing the R359K mutation from an MD simulation sampled immediately after in silico-transformation of ATP into ADP and P_i_ for 2 μs. K359 is positioned in between ADP and the cleaved P_i_ ion, symmetrically opposite to K251 (c.f. Extended Data Fig. 8b). In contrast to the wt protein, only a single ADP·P_i_ geometry is observed, which is very stable (c.f. Fig. 5a) and similar to state A with respect to the positioning of the Mg^2+^ and P_i_ ions and the dissociation of F360 from helix α_407–423_. The R359K mutant does not release the reaction products efficiently.

Supplementary Video 3 Close-up of the D1 active site with F360P mutation from an MD simulation sampled immediately after in silico-transformation of ATP into ADP and P_i_ for 2 μs. The P_i_ and Mg^2+^ ions move between positions that are similar to ADP·P_i_ states A and B of the wt. However, the absence of the F360 side chain prevents the coupling of ATP-hydrolysis events to dissociation from helix α_407–423_.

Supplementary Video 4 Close-up of the D1 nucleotide binding pocket in the apo state in the presence of exogeneous P_i_ ions. The ADP·P_i_ state is mimicked by two P_i_ ions bridged by K^+^ ions from the solvent (green), which exchange frequently during the 1-μs simulation time. The P_i_ ions occupy the same positions as the β-P of ADP and the cleaved P_i_ ion. A Mg^2+^ ion is not required for stabilization of the arrangement.

Supplementary Video 5 The NTD moves upon ATP hydrolysis in D1, starting from the ‘up’ position observed in the ATPγS state (PDB 5FTN)^5^ to the ‘down’ position in ADP·P_i_ states A and B (PDB 8OOI, this work). The active site is located at the inter-subunit interface, with the *cis*-acting subunit shown in purple and the *trans*-acting in pink. The transition between ADP·P_i_ states A and B is evident in rotamer switches of R359 and F360 and repositioning of the P_i_ and Mg^2+^ ions. Free-energy calculations (c.f. Fig. 5a) suggest that state B marks the onset to dissociation. H384 is located next to the ribose moiety of the nucleotide and displays two side-chain rotamers in the ADP·P_i_ state, which cannot be assigned to states A or B with certainty. In the NTD ‘down’ position, electrostatic interactions, which form only after ATP hydrolysis, stabilize the NTD-D1 interface: between E34 and K386 as well as between R155 and N387.

### Source data

Source Data Fig. 2 Source data for the MD simulation.

Source Data Fig. 3 Statistical source data for ATPase rates.

Source Data Fig. 4 Source data for the MD analysis.

Source Data Fig. 5 Statistical source data for the MMBSA calculations and ATPase rates.

Source Data Fig. 6 Source data for the MD simulation.

Source Data Extended Data Fig./Table 4 Statistical source data for ATPase rates.

Source Data Extended Data Fig./Table 6 Source data for the MD simulation.

Source Data Extended Data Fig./Table 8 Statistical source data for the MMBSA calculations and ATPase rates.

## Figures and Tables

**Extended Data Fig. 9:**
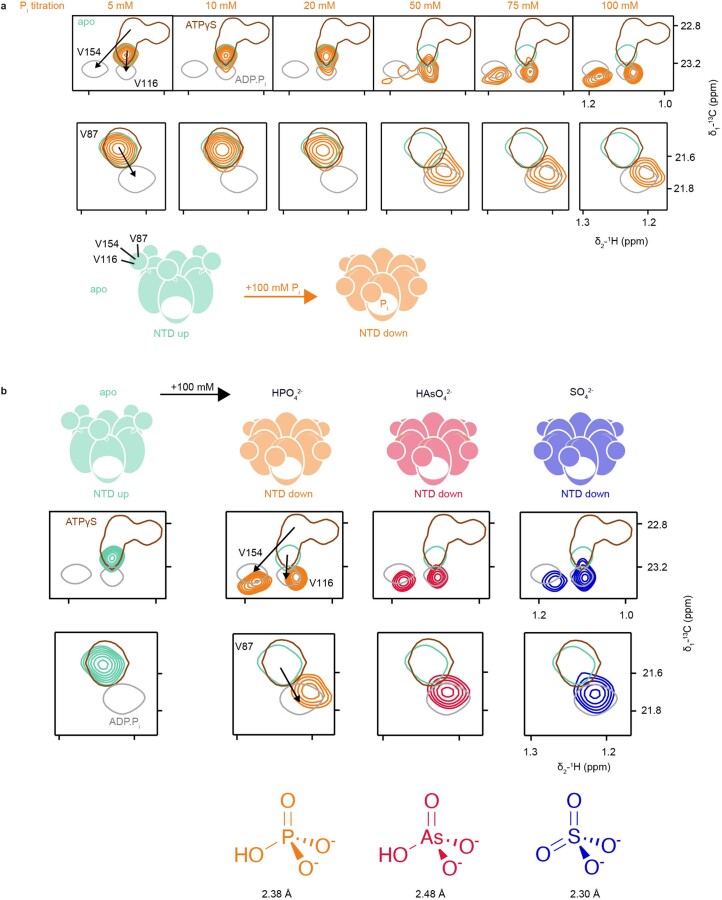
Structural effect of P_i_ ions and mimics on apo p97. **a**, A concentration of ~75 mM inorganic P_i_ ions in solution induces a complete movement of the NTD in apo p97-ND1L into the ‘down’ position. Residues V116, V154 and V87 report on the NTD position. The arrows indicate the shifts of representative methyl correlations from the ‘up’ position (ATPγS state as reference in single contours) to ‘down’ position (ADP·P_i_ state as reference). Mutants P247K, R359A and R362A do not form hexamers in apo state and do not respond to Pi addition (Supplementary Fig. 12). Note that fl p97 requires a threefold higher P_i_ concentration to achieve even a partial effect on NTD position. **b**, Top: Arsenate (HAsO_4_^2−^) and sulphate (SO_4_^2−^) can mimic phosphate (HPO_4_^2−^) at a concentration of ~100 mM. Bottom: Comparison of thermochemical radii of these ions at pH 7.5 as an estimate of their effective size in solution^78^. In the light of our observations, a crystal structure of apo state p97 where the NTD is found in the ′down′ state^79^ may be attributed to a sulphate ion trapped between P247 and K251.

## Data Availability

Data supporting the findings of this work are available within the Article, Extended Data, Supplementary Information and source data files. Further details and raw data from in silico modelling are also available from the corresponding authors upon request. Cryo-EM maps, model coordinates and associated structure factors of p97 in the ADP·P_i_ state have been deposited in the Electron Microscopy Data Bank (EMDB; EMD-16781 (NTD-focused maps), EMD-17016 (full p97 composite map), EMD-17024 (D1–D2 focused map) and EMD-17128 (consensus map)) and Protein Data Bank database (PDB 8OOI). The publicly available datasets used can be found under PDB accessions 3HU1, 3HU2, 3HU3, 4KO8, 5C1A, 5FTK, 5FTL, 5FTM, 5FTN, 7JY5, 7LMY, 7LMZ, 7LN0, 7LN1, 7LN2, 7LN3, 7LN4, 7LN5, 7RLA, 7RLC, 7RLF, 7RLH, 7RLJ, 7RL7, 7VCS, 7VCT, 7VCU, 7VCV and 7VCX. Source data are provided with this paper.
