# Supplementary material for: Characterizing ATP processing by the AAA+ protein p97 at the atomic level
Source: Nat Chem. 2024 Feb 7;16(3):363–72. doi: 10.1038/s41557-024-01440-0 (PMC10914628; doi:10.1038/s41557-024-01440-0)
Supplement: Supplementary file 1 — Supplementary information, including references, Figs. 1–13 and Tables 1–8. [file 41557_2024_1440_MOESM1_ESM.pdf]

nature chemistry

Article

https://doi.org/10.1038/s41557-024-01440-0

Characterizing ATP processing by the AAA+ protein p97 at the atomic level

In the format provided by the authors and unedited

Table of contents

Supplementary Figures 2

Supplementary Fig. 1 Flow chart of cryo-EM data analysis. 2

Supplementary Fig. 2 Cryo-EM image processing of p97 in the ATP regeneration system. 3

Supplementary Fig. 3 FSC curves of cryo-EM reconstructions. 4

Supplementary Fig. 4 Atomic-level characteristics of the ADP.Pi state. 6

Supplementary Fig. 5 Sequence alignment of p97 in different eukaryotic organisms. 7

Supplementary Fig. 6 NMR spectra of p97-ND1L K251A. 8

Supplementary Fig. 7 NMR spectra of p97-ND1L D304N. 9

Supplementary Fig. 8 NMR spectra of p97-ND1L F360P. 10

Supplementary Fig. 9 NMR spectra of p97-ND1L N348Q. 11

Supplementary Fig. 10 Fraction of reaction-competent conformations. 12

Supplementary Fig. 11 Conformational changes in the sensor loop. 13

Supplementary Fig. 12 Comparison of point mutants in apo state. 14

Supplementary Fig. 13 Crosslinking of the ΔCys-F360C-A413C mutant. 15

Supplementary Tables 16

Supplementary Table 1. Experimental setup for solution-state NMR data acquisition. 16

Supplementary Table 2. Experimental setup for solid-state NMR data acquisition. 16

Supplementary Table 3. Cryo-EM data collection and processing statistics. 17

Supplementary Table 4. Cryo-EM model building. 18

Supplementary Table 5. Overview of all MD simulations. 19

Supplementary Table 6. Overview of the seven equilibration steps performed for all simulations. ... 19

Supplementary Table 7. Binding affinities of p97-ND1L wt and mutants towards ADP and ATPγS determined by ITC 20

Supplementary Table 8. Reference structures used in violin plot analysis. 21

Supplementary References 22

Supplementary Figures

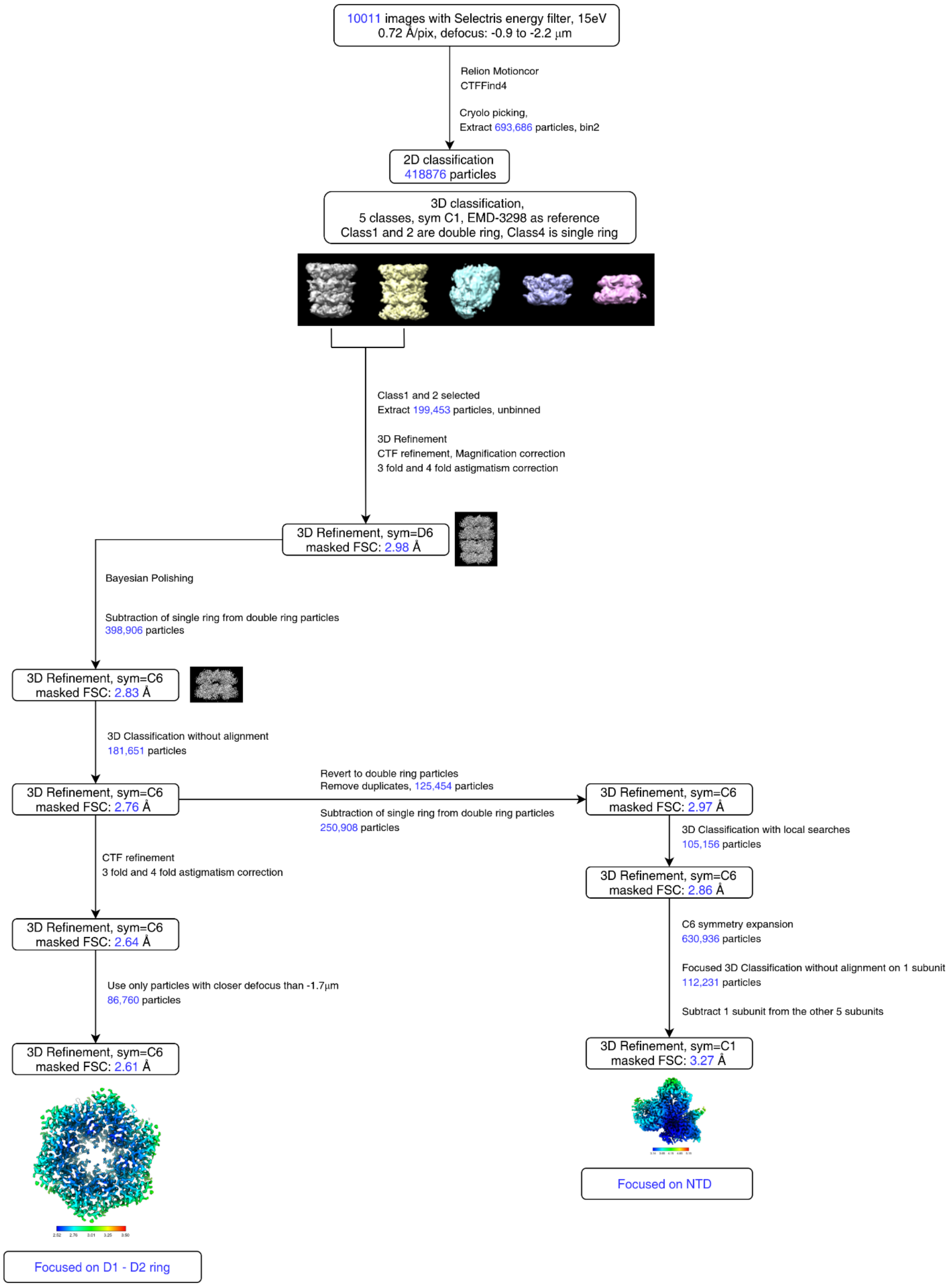

Supplementary Fig. 1 Flow chart of cryo-EM data analysis. Reconstruction of wt fl p97 in the presence of an ATP-regeneration system. Classification and refinement procedures for the D1-D2 focused maps and NTD focused maps are shown. Briefly, single ring hexamers were computationally subtracted from double ring hexamers, followed by cycles of focused 3D classification, CTF refinements and focused refinements with C6 symmetry applied. Adding single ring hexamers into the mix resulted in inferior resolution (2.9 Å, data not shown), possibly because D2 domain in double ring hexamers is more stabilized than that of single ring hexamers. Since NTD densities are often too fragmented in symmetrized p97 reconstructions, focused classification and refinement were performed to improve the NTD densities.

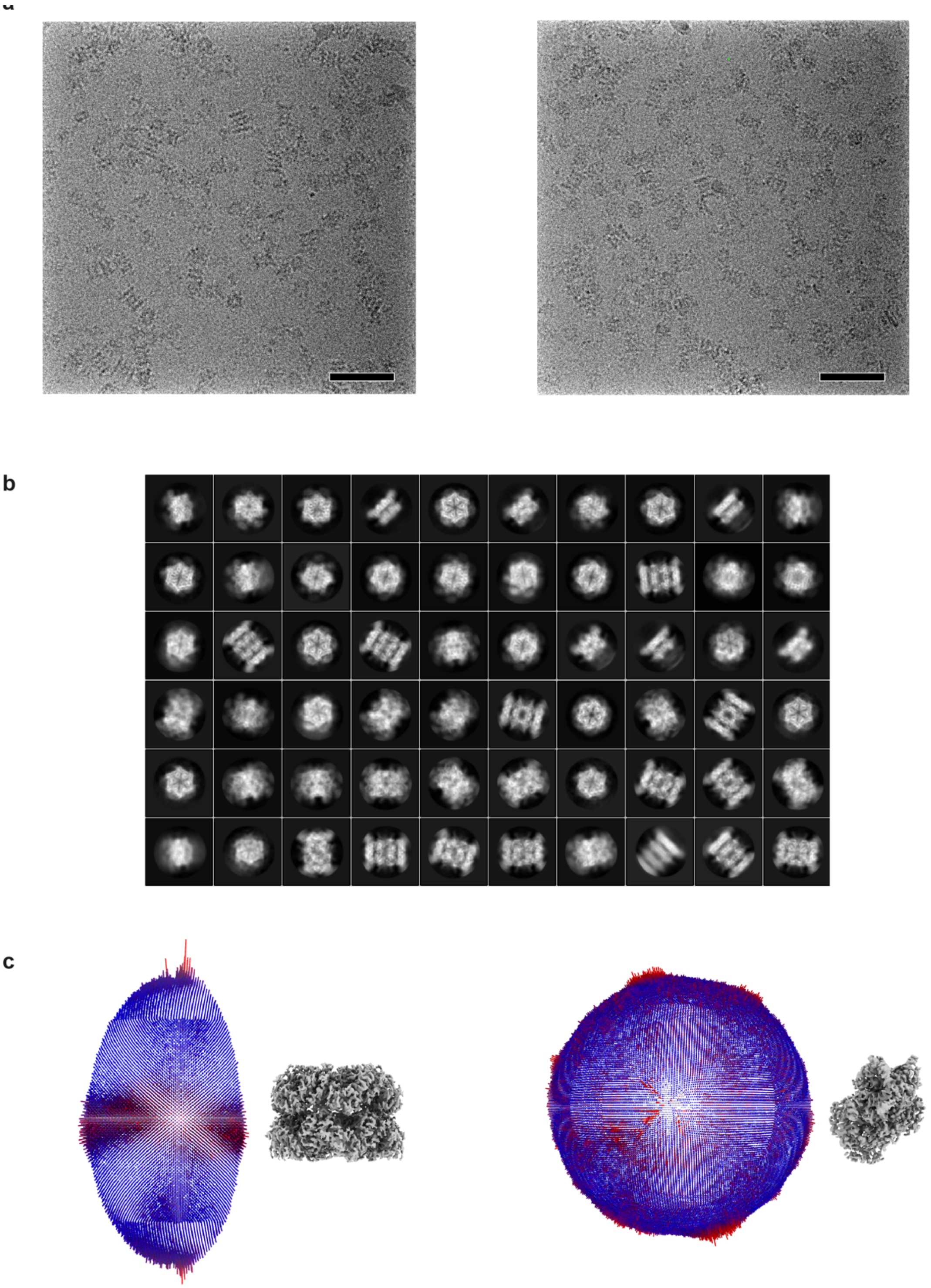

Supplementary Fig. 2 Cryo-EM image processing of p97 in the ATP regeneration system. **a**, Representative micrographs of wt p97 in the presence of an ATP-regeneration system, scale bar represents 50 nm. These two micrographs are representative of a vast majority (98%) of the whole dataset in terms of particle number, particle distribution and ice thickness. **b**, Two-dimensional class averages. **c**) Angular distribution of particle views for the reconstruction with focus on the D1-D2 ring (left) and with focus on the NTD (right).

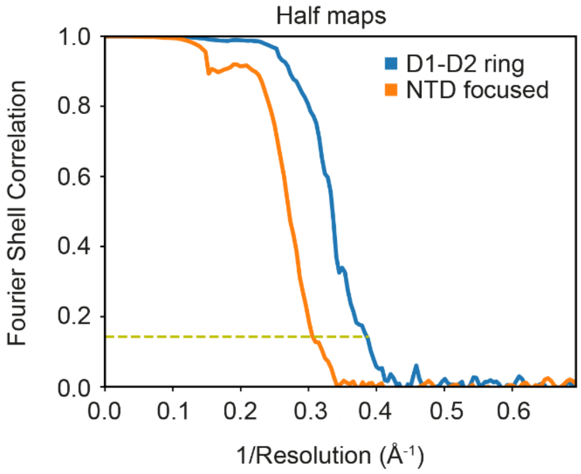

Supplementary Fig. 3 FSC curves of cryo-EM reconstructions. Resolutions were determined on the basis of the gold standard Fourier shell correlation between two independently refined half maps[1] (FSC=0.143, dotted line). The D1-D2 ring focused map has a resolution of 2.61 Å compared to 3.27 Å for the NTD focused map.

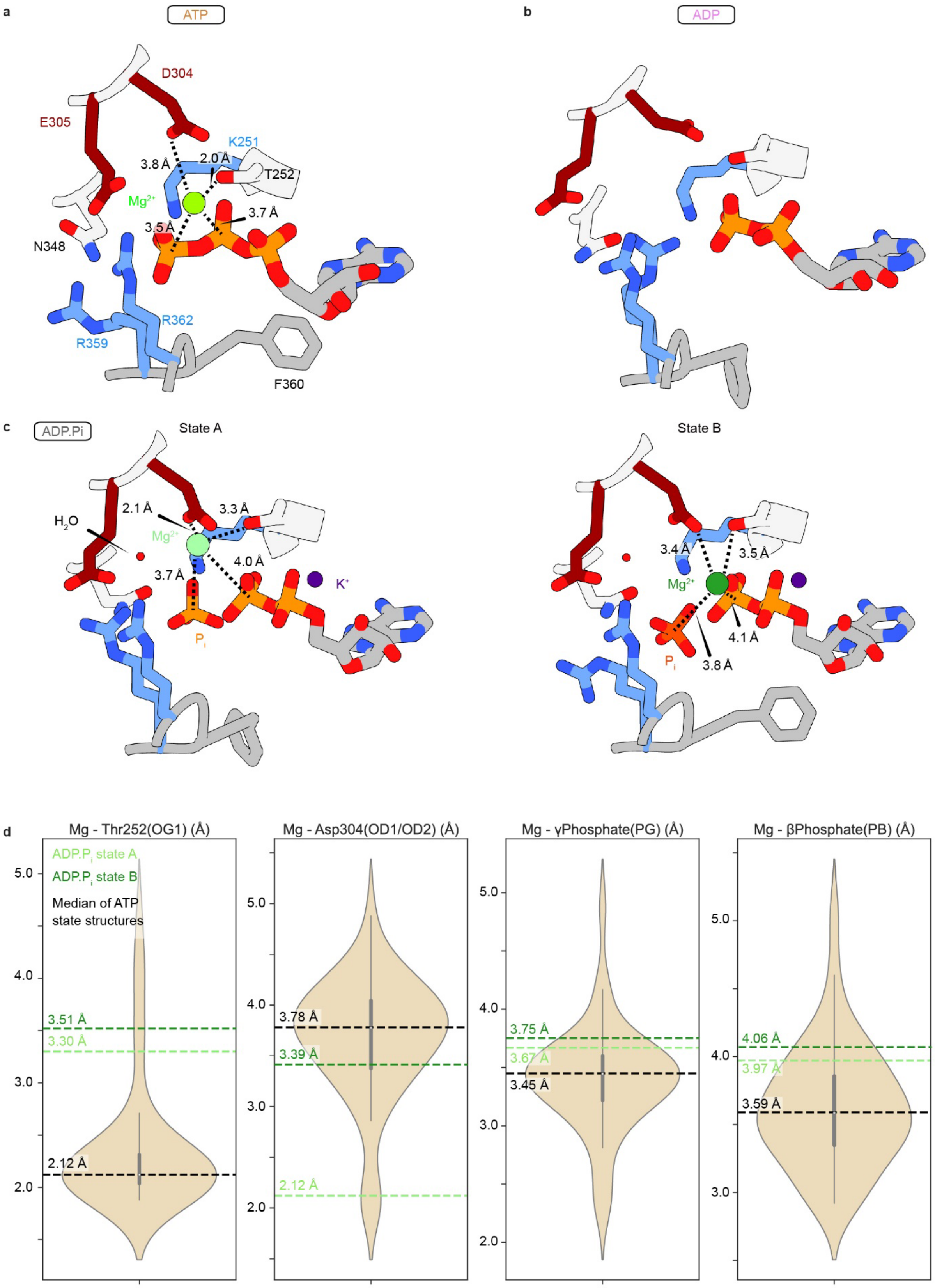
a
ATP
D304
E305
K251
3.8 Å
2.0 Å
T252
Mg2+
3.7 Å
3.5 Å
N348
R362
R359
F360
b
ADP
c
ADP.Pi
State A
State B
H2O
2.1 Å
3.3 Å
Mg2+
4.0 Å
3.7 Å
K+
Pi
3.4 Å
3.5 Å
4.1 Å
3.8 Å
d
Mg - Thr252(OG1) (Å)
Mg - Asp304(OD1/OD2) (Å)
Mg - γPhosphate(PG) (Å)
Mg - βPhosphate(PB) (Å)
ADP.Pi state A
ADP.Pi state B
Median of ATP state structures
3.51 Å
3.30 Å
2.12 Å
3.78 Å
3.39 Å
2.12 Å
3.75 Å
3.67 Å
3.45 Å
4.06 Å
3.97 Å
3.59 Å

Supplementary Fig. 4 Atomic-level characteristics of the ADP.Pi state.

a-c, Juxtaposition of the D1 nucleotide binding pocket in ATPγS (PDB: 7lmy[2]), ADP (PDB: 5ftk[3]) and ADP.P_i states (PDB: 8ooi, this work) with interatomic distances of the Mg^{2+} ion to T252, D304, β- and γ-phosphate moieties of the bound nucleotide or the cleaved P_i ion, respectively. After ATP cleavage, the Mg^{2+} ion moves away from T252 and closer to D304. **d,** Distance measurements were performed on 44 published X-ray crystallography and cryo-EM structures of p97, in which the nucleotide state in D1 was assigned as ATP or ATPγS (listed in Supplementary Tab. 8). The results are illustrated as violin plots. The median distance of all published structures is marked black in each plot. The thick grey line indicates the 1st and 3rd quartiles of the underlying box plot, the thin grey lines indicate its whiskers. The distances in our model of the ADP.P_i state are marked light (state A) and dark green (state B). The ADP.P_i states capture chemical environments in the D1 pocket that differ from the published structures of the ATP-bound state. In most of ATP-bound structures, Mg^{2+} stably coordinates T252 and the β- and γ-phosphate moieties, while D304 remains unengaged. In contrast, in both ADP.P_i states, Mg^{2+} has dissociated from T252. In state A, the Mg^{2+} ion is in close proximity to D304, but in state B it has moved away. Additionally, in the ADP.P_i states, the Mg^{2+} ion moves away from the β-phosphate but remains associated. In both states A and B, we observe that the Mg^{2+} ion pairs with the cleaved P_i ion**.**

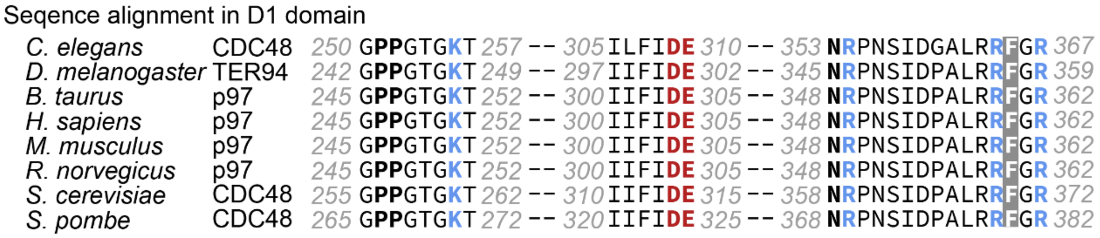

Supplementary Fig. 5 Sequence alignment of p97 in different eukaryotic organisms. The phenylalanine residue between the arginine fingers in D1 (F360 in human p97) is a special feature not found in other eukaryotic AAA+ proteins (cf. Fig. 3a). While it is strictly conserved among p97 homologues, this position is often occupied by a proline residue in AAA+ proteins.

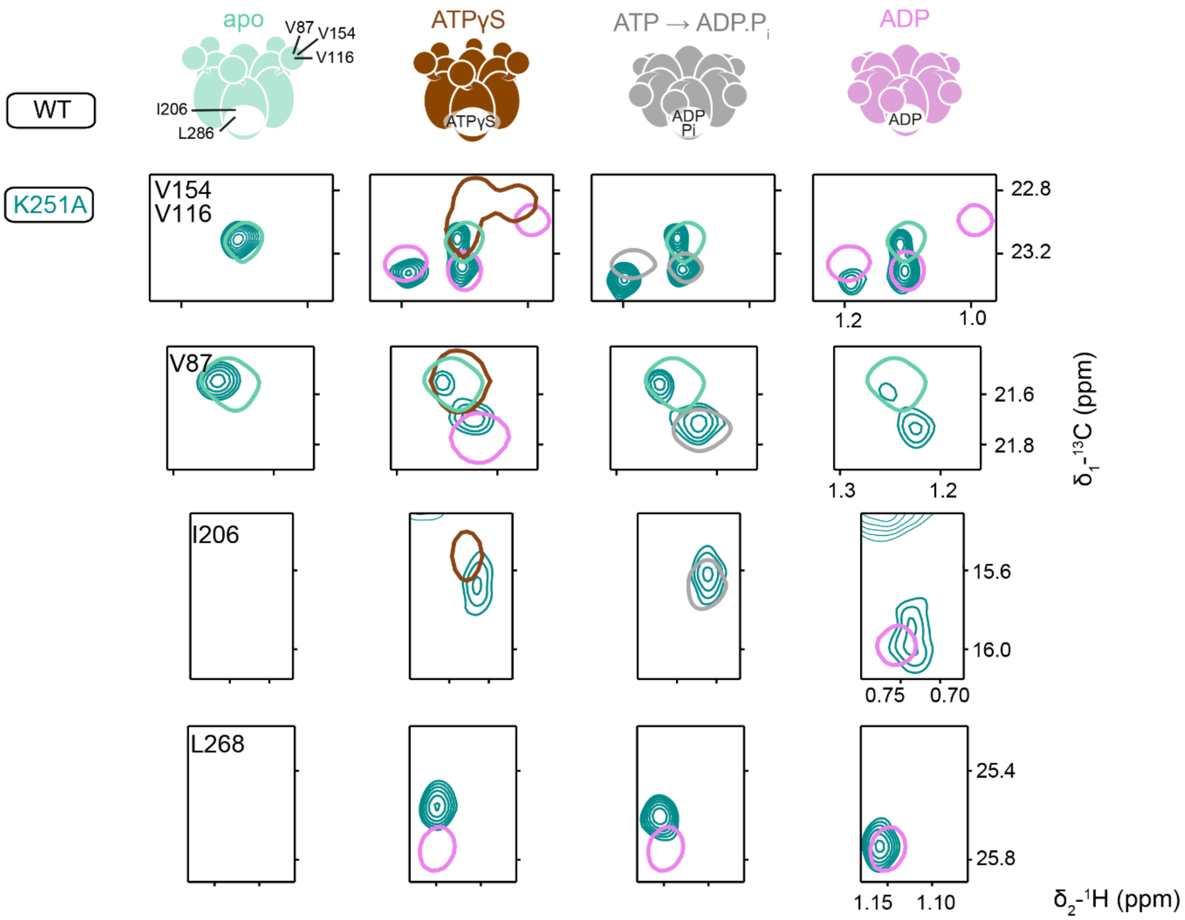

Supplementary Fig. 6 NMR spectra of p97-ND1L K251A. Selected spectral regions from (^{1}H,^{13}C)-HMQC spectra of p97-ND1L K251A as a function of nucleotide present. Residues V116, V154 and V87 report on the NTD position, while residues I206 and L268 reflect the conformation of the D1 binding pocket. Methyl group correlations from the respective wt spectra are indicated in single contours; when wt and mutant spectra differ, the corresponding wt-ADP spectra are shown in addition. Although the mutant lacks the K251 residue that is critical for nucleotide binding[4], the NMR spectra are still sensitive to the presence of nucleotide and indicate a mixture of NTD 'up' and 'down' states. The NMR data thus indicate that the D1 binding pocket is still able to interact with nucleotide but not to structurally discriminate ATP(γS) from ADP. The binding affinity of K251A mutant for ADP is below the detection limit of ITC (Supplementary Table 7).

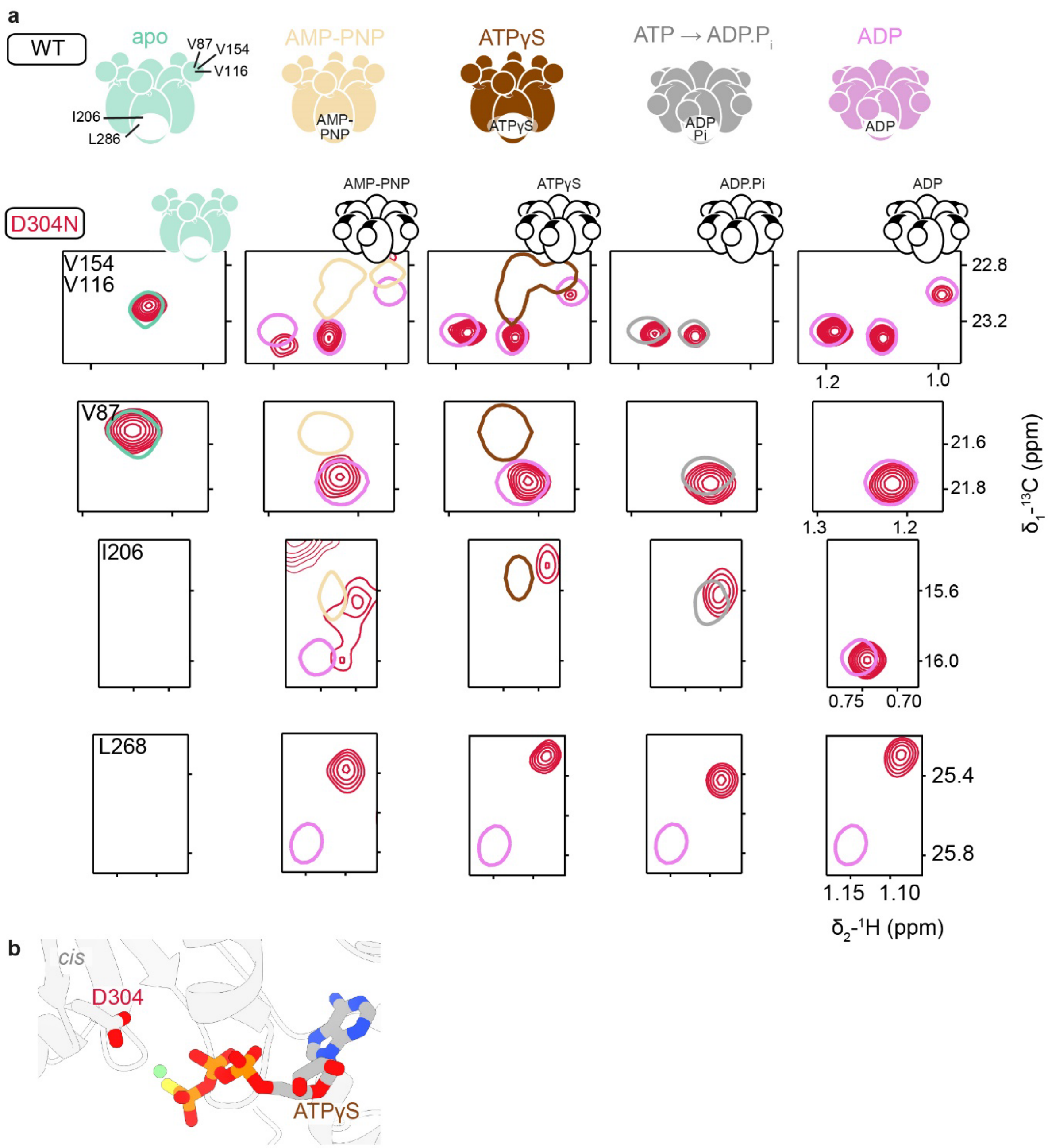

Supplementary Fig. 7 NMR spectra of p97-ND1L D304N. a, Selected spectral regions from (^{1}H,^{13}C)-HMQC spectra of p97-ND1L D304N as a function of nucleotide present. Residues V116, V154 and V87 report on the NTD position, while residues I206 and L268 reflect the conformation of the D1 binding pocket. Methyl group correlations from the respective wt spectra are indicated in single contours; when wt and mutant spectra differ, the corresponding wt-ADP spectra are shown in addition. **b**, Close-up of the D1 binding pocket in ATPγS state. D304 is crucial for Mg^{2+} binding. The D304N mutant binds nucleotide (*cf.* Supplementary Table 7 and NMR signals of I206/L268 in nucleotide presence *vs.* absence), yet it fails to assume an NTD ‘up’ state in the presence of ATPγS/AMP-PNP. The coordination of Mg^{2+} is a prerequisite to structurally recognize ATP and its analogues. Due to its low ATPase rate (Fig. 3b), it remains unclear whether the D304N mutant is able to hydrolyse ATP at all.

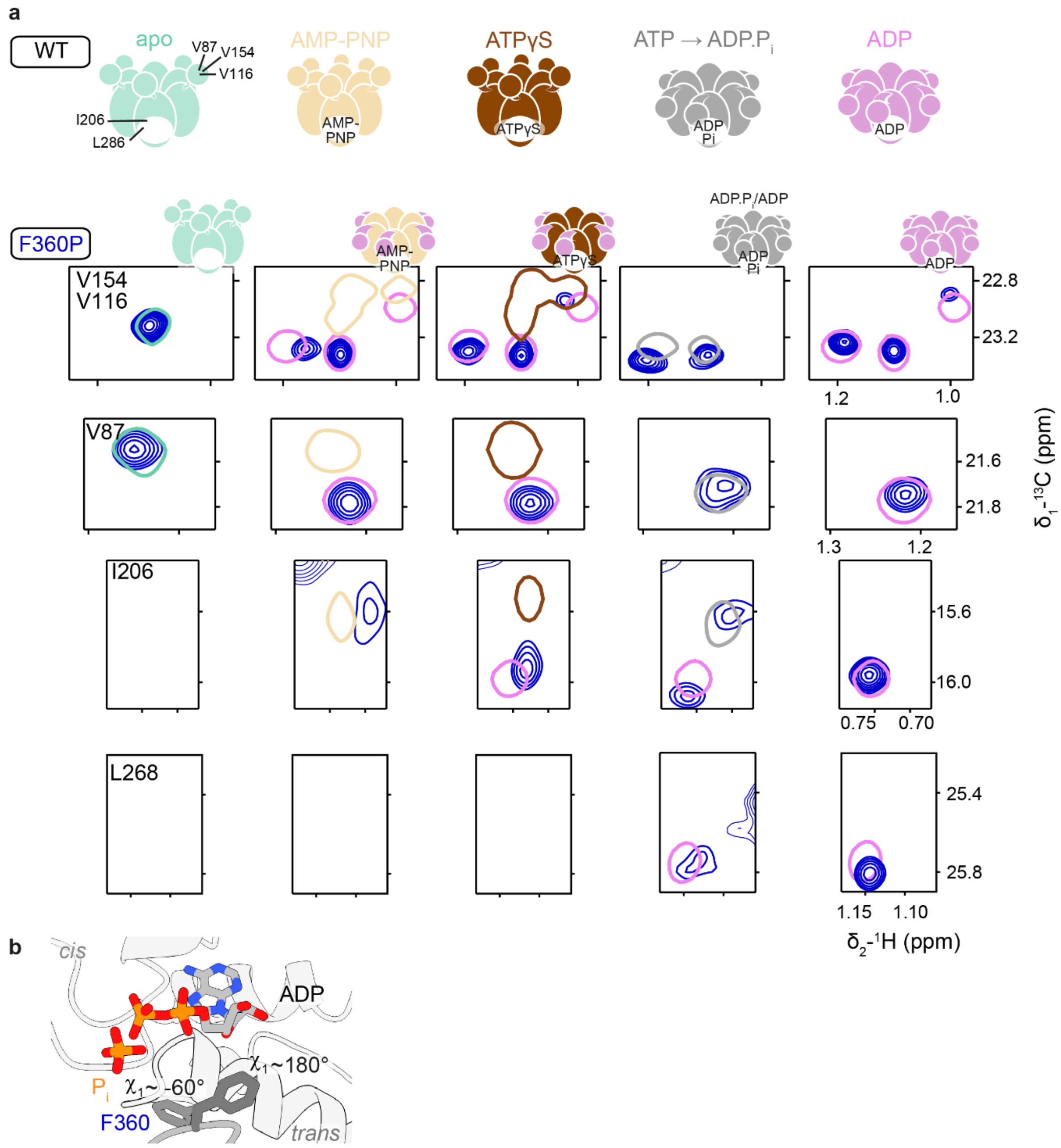

Supplementary Fig. 8 NMR spectra of p97-ND1L F360P. a, Selected spectral regions from (^{1}H,^{13}C)-HMQC spectra of p97-ND1L F360P as a function of nucleotide present. Residues V116, V154 and V87 report on the NTD position ('up' *vs*. 'down'), while residues I206 and L268 reflect the conformation of the D1 binding pocket and its nucleotide. Methyl group correlations from the respective wt spectra are indicated in single contours; when wt and mutant spectra differ, the corresponding wt-ADP spectra are shown in addition. **b**, Close-up of the D1 binding pocket in ADP.P_i state. F360 switches between different rotamers: χ_1~180° allows for association with the neighbouring helix $\alpha_{407-423}$, while χ_1~-60° leads to disassociation. The F360P mutation decouples the NTD position from the D1 nucleotide state, evidenced by the NTD 'down' position in the presence of AMP-PNP and ATPγS. Spectra recorded in the presence of ATP show a mixture of pre-hydrolysis (AMP-PNP-like) and post-hydrolysis (ADP-like) states for residue I206.

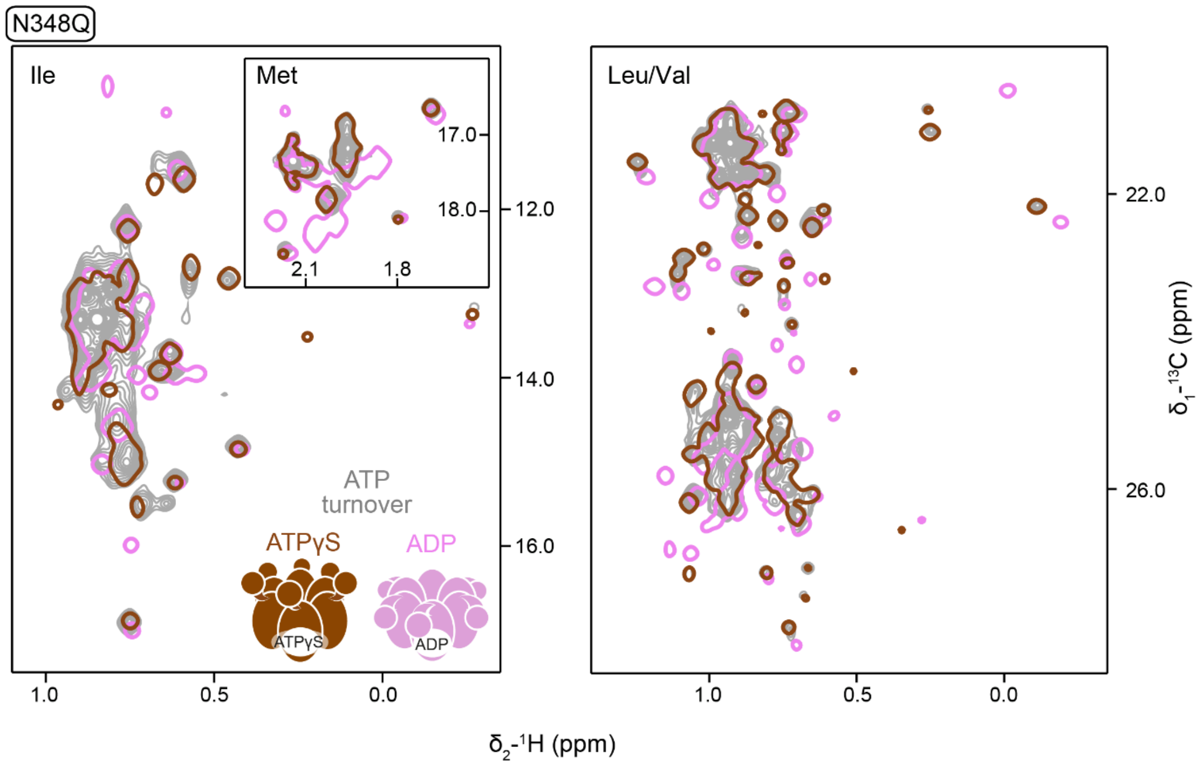

Supplementary Fig. 9 NMR spectra of p97-ND1L N348Q. (^{1}H,^{13}C)-HMQC spectra of *proR*-$^{13}CH_3$-ILVM-labelled p97-ND1L N348Q. The spectrum recorded in the presence of the ATP-regeneration system (multiple grey contours) is very similar to the spectrum recorded in the presence of slowly-hydrolysable ATPγS (single brown contours), documenting the inability of the N348Q mutant to hydrolyse ATP.

Fraction of active conformations [%]

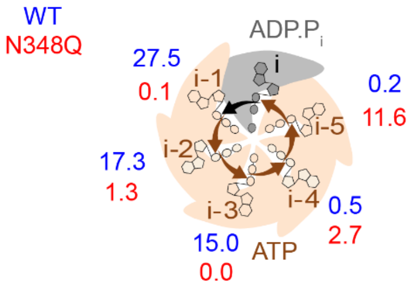

Supplementary Fig. 10 Fraction of reaction-competent conformations. Conformations poised for ATP hydrolysis as defined in Fig. 3e were identified at each ATP-bound D1 active site over all frames of the full MD simulation of the wt protein and the N348Q mutant. Reaction-competent conformations are found in a significantly higher number of frames for the wt protein than for the mutant. The single ADP.P_i-bound subunit breaks the symmetry of the hexamer. In the simulation of the wt, the fraction of reactive conformations in each subunit declines from the ADP.P_i-bound subunit in a counter clockwise manner. This distribution is reminiscent of the unidirectional hydrolysis mechanism postulated for many AAA proteins based on cryo-EM structures[5]. However, testing the significance of this observation will require more extensive statistics.

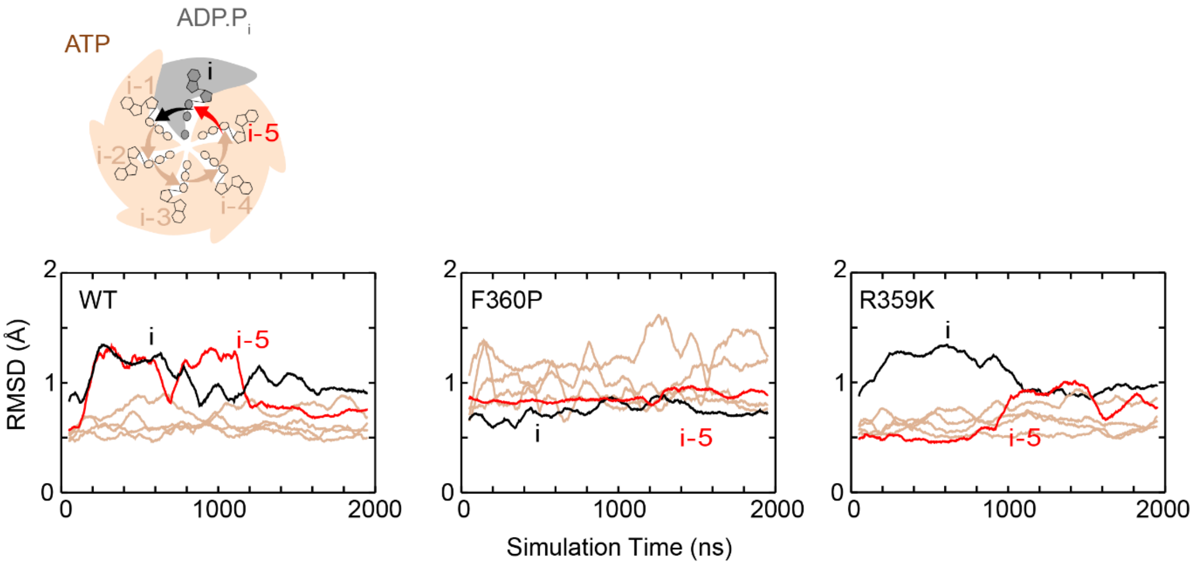

Supplementary Fig. 11 Conformational changes in the sensor loop. RMSD (root-mean-quare deviation) fluctuations indicate the deviation of the backbone of sensor loop residues (348-361) from their position in the first frame over the course of the 2 µs-MD trajectory. In the wt protein, the loop displays increased mobility if one of the two adjacent active sites is occupied by ADP.P$_i$ (red/black) compared to ATP (brown). In the hyperactive F360P mutant, the mobility of the loop is overall increased, irrespective of nucleotide state. In the inactive R359K mutants, loop mobility is overall decreased.

a

Mutant	wt	P246T	P247A	P247K	K251A	D304N	E305Q	N348Q	R349A	R359A	R359K	F360A	F360P	R362A
NTD down motion upon P_i binding	v	x	v	~	~	v	~	v	v	~	v	v	v	x
Hexamer in apo state	v	v	v	x	v	v	~	v	v	x	v	v	v	x

v	yes
x	no
~	partial

b

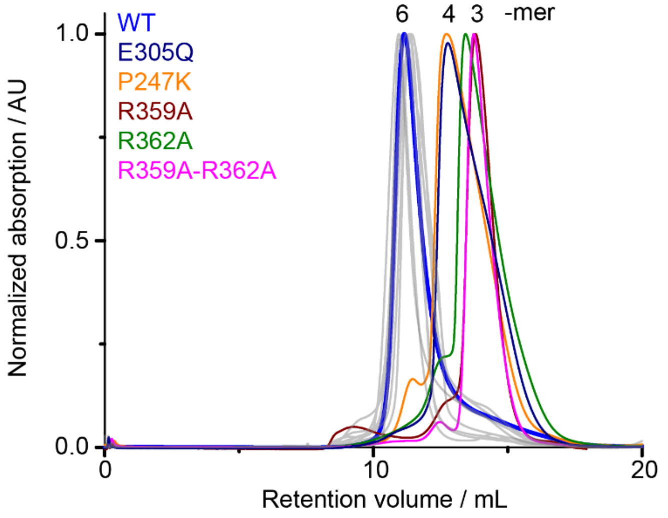

Supplementary Fig. 12 Comparison of point mutants in apo state. a, Structural response of p97-ND1L mutants to P_i ion addition and oligomeric states of the respective mutants in the absence of nucleotide. The response of a given mutant to P_i binding is linked to its ability to form intact hexamers in apo state. **b**, The oligomeric states of p97-ND1L mutants in the absence of nucleotide were assessed by SEC. Non-hexameric mutants and the wt are highlighted in colour, all others are shown in grey. P274K and R359A require nucleotide for hexamerisation, R362A mutants do not form hexamers at all (Extended Data Fig. 4).

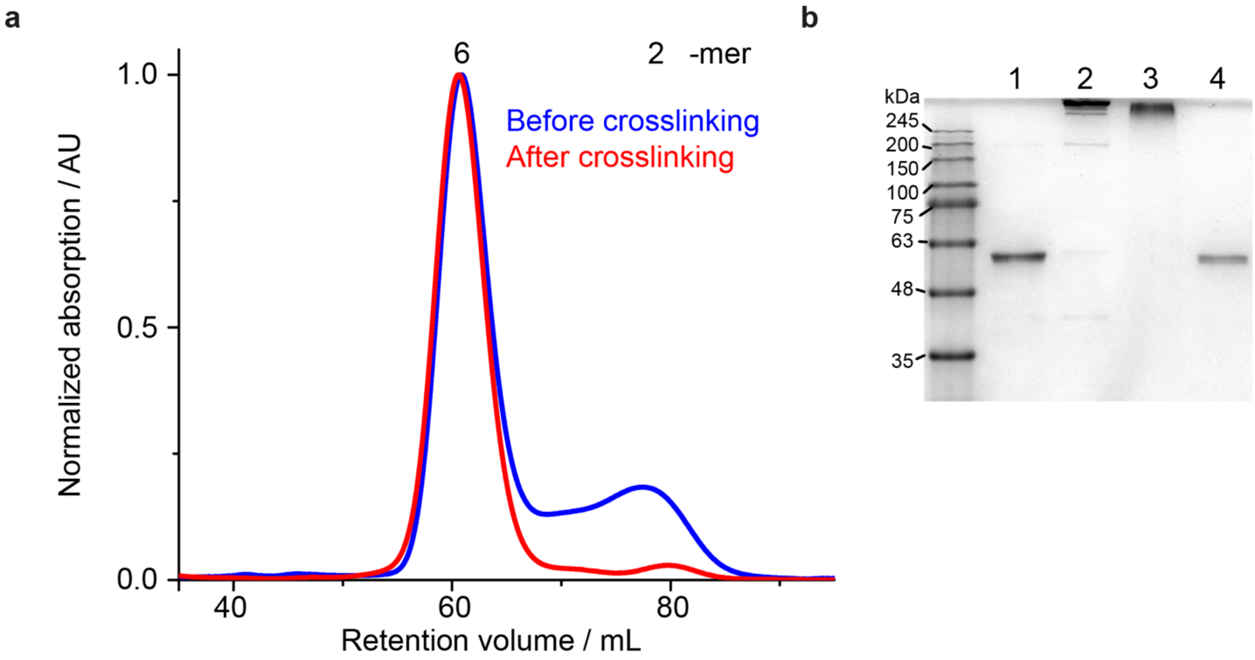

Supplementary Fig. 13 Crosslinking of the ΔCys-F360C-A413C mutant.

a, Oligomeric state of the p97-ND1L ΔCys-F360C-A413C mutant assessed by SEC before and after crosslinking with BMOE. In both cases the protein elutes at a volume corresponding to a hexameric assembly. **b**, SDS-PAGE confirming the crosslinking. 1: peak from SEC corresponding to hexameric p97 before crosslinking; 2: crosslinking reaction mixture; 3: peak from SEC corresponding to hexameric p97 after crosslinking; 4: ND1L wt reference. The crosslinking experiment was performed twelve times independently with four biologically independent samples with similar results.

Supplementary Tables

Supplementary Table 1. Experimental setup for solution-state NMR data acquisition.

Spectrometer ^{1}H frequency [MHz]	800, 900, 950
Probe	cryo-TCI
Pulse sequence	(^{1}H,^{13}C)-HMQC
Number of scans	4
Acquisition time [min]	42
Spectral width [ppm]	16.0(^{1}H) / 25.0(^{13}C)
Transmitter frequency offset [ppm]	4.707(^{1}H) / 18.6(^{13}C)

Supplementary Table 2. Experimental setup for solid-state NMR data acquisition.

Spectrometer ^{1}H frequency [MHz]	800	
Probe	Bruker 1.3 mm WB in HPC mode	
MAS rate	45 kHz	
Pulse sequence	Direct excitation	Cross-polarization
Description	^{31}P direct excitation spectra, ^{1}H decoupling during acquisition	^{1}H-^{31}P cross-polarization spectra, ^{1}H decoupling during acquisition
90° excitation pulse [µs]	2.0 (^{31}P)	1.59 (^{1}H)
Cross-polarization transfer		Ramp 70-100%, 30 kHz (^{31}P), 93 kHz (^{1}H)
Transmitter frequency offset [ppm]	0 (^{31}P), 4.0 (^{1}H)	0 (ATP) / 15 (ATPγS) (^{31}P), 4.0 (^{1}H)
^{1}H decoupling during acquisition	slTPPM, 11 kHz	
Acquisition time [ms]	41 (^{31}P)	20.5 (^{31}P)
Number of scans/ experimental time [min]	512/69	8192/140
Spectral width [ppm]	154.3	
Interscan delay [s]	8	1

Supplementary Table 3. Cryo-EM data collection and processing statistics.

**Sample**	fl p97 in ATP-regenerating buffer system			
**Data Collection**				
Microscope		Titan Krios		
Voltage (kV)		300		
Magnification		165,000		
Pixel size (Å)		0.72		
Exposure rate (e/Å^2/s)		10.4		
Total Exposure (e/Å^2)		40		
Nominal defocus range (μm)		-0.9 to -2.2		
Energy filter and detector		Selectris and Falcon 4		
Energy filter slit (eV)		15		
Number of images		10011		
Number of EER frames		931		
**Image Processing**				
Name of the maps	Full composite map	Consensus map	D1-D2 Focused	NTD Focused
Symmetry imposed		C6	C6	C1
Initial particle number		693,686	693,686	693,686
Final particle number		53,024	86,760	112,231
FSC threshold		0.143	0.143	0.143
Map resolution (Å)		3.05	2.61	3.27
Map resolution range (Å)		2.82 – 5.46	2.53 – 4.03	3.14 – 5.41
Map sharpening B factor (Å^2)		-74	-58	-67
EMDB entry	EMD-17016	EMD-17128	EMD-17024	EMD-16781

Supplementary Table 4. Cryo-EM model building.

**Model statistics**	
Initial model used (PDB code)	5ftl, 5ftm[3]
Nonhydrogen atoms	35334
Chains	6
Protein residues	4404
Water	246
Ligands	K^+: 6, PO_4^{3-}: 6, Mg^{2+}: 12, ATP: 6, ADP: 6
R.m.s deviations<br>Bond length (Å)<br>Bond angles (°)	 0.0048<br>0.70
Ramachandran plot<br>Favored (%)<br>Allowed (%)<br>Outliers (%)	 95.05<br>4.54<br>0.41
Rotamer outliers (%)	0.16
Clashscore	10.47
Molprobity score[6]	1.88
PDB entry	8ooi

Supplementary Table 5. Overview of all MD simulations. All simulations were performed on p97 hexamers.

Simulation Name	System	Template	Sampling time
**ADP.P_i prediction**	ND1L, 5xATP, 1xADP.P_i (HPO_4^{2-})	4ko8	2000 ns
**ADP.P_i prediction**	ND1L, 5xATP, 1xADP.P_i ($H_2PO_4^-$)	4ko8	1000 ns
**R359K mutant**	ND1L, 5xATP, 1xADP.P_i, R359K	4ko8	2000 ns
**F360P mutant**	ND1L, 5xATP, 1xADP.P_i, F360P	4ko8	2000 ns
**N348Q mutant**	ND1L, 5xATP, 1xADP.P_i, N448Q	4ko8	2000 ns
**P_i + P_i**	ND1L, 5xATP, 1xP_i+P_i, no Mg^{2+}	4ko8	1000 ns
**ADP.P_i refinement**	D1-D2, 6xADP.P_i (D1), 6xATP (D2)	This work	1300 ns
**P_i dissociation**	Like above, no Mg^{2+}	This work	700 ns

Supplementary Table 6. Overview of the seven equilibration steps performed for all simulations. The initial minimization is followed by six equilibration simulations with decreasing force constants (K) on positional restraints of amino acid residues, ATP, ADP, P_i and Mg^{2+} ions.

	Steps	Time step	K solute	Temp.	Pressure
**min**	1000	minimization	10.0	---	---
**Eq1**	25000	1fs	10.0	303.15 K	NA
**Eq2**	25000	1fs	5.0	303.15 K	NA
**Eq3**	25000	1fs	2.5	303.15 K	1 atm
**Eq4**	50000	2fs	1.0	303.15 K	1 atm
**Eq5**	50000	2fs	0.5	303.15 K	1 atm
**Eq6**	250000	2fs	0.1	303.15 K	1 atm

Supplementary Table 7. Binding affinities of p97-ND1L wt and mutants towards ADP and ATPγS determined by ITC. Abbreviations: n.d. no binding detected; n number of replicates. In the absence of Mg^{2+} ions, p97 cannot bind ATPγS with high affinity. This experiment serves as a negative control.

p97-ND1L	ADP K_D (μM)	ATPγS K_D (μM)
WT	0.08 ± 0.01 (n=3)	0.19±0.07 (n=3)
P246T	0.02 ± 0.0007 (n=2)	0.09 ± 0.05 (n=3)
P247A	0.08 ± 0.02 (n=3)	0.18 ± 0.16 (n=2)
P247K	0.05 ± 0.03 (n=3)	0.01 ± 0.004 (n=2)
D304N	0.05 ± 0.002 (n=2)	0.30 ± 0.08 (n=3)
E305Q	0.32 ± 0.001 (n=2)	0.08 ± 0.001 (n=3)
N348Q	0.18 ± 0.04 (n=3)	0.68 ± 0.06 (n=2)
R359A	1.35 ± 0.78 (n=3)	0.04 ± 0.02 (n=3)
R359K	0.11 ± 0.03 (n=3)	0.21 ± 0.16 (n=3)
F360A	0.06 ± 0.03 (n=3)	0.23 ± 0.01 (n=2)
F360P	0.17 ± 0.02 (n=3)	0.14 ± 0.02 (n=2)
R359A-R362A	0.05 ± 0.03 (n=3)	0.11 ± 0.02 (n=2)
K251A	n.d. (n=2)	
wt without Mg^{2+}		1.08 ± 0.007 (n=2)

Supplementary Table 8. Reference structures used in violin plot analysis. Subunits in nucleotide states other than ATP or ATPγS were not included in the statistics of the ATP-state reference structures.

PDB ID	ATP or ATPγS	Mg-γP		Mg-βP		Mg-αP		Mg-T252	Mg-E304	K251-γP
Distance to atom type		Mg-P	Mg-O	Mg-P	Mg-O	Mg-P	Mg-O	Mg-O	Mg-O	N-P
This publication (ADP.P$_i$ state A)		3.67	2.39	3.97	4.10	5.88	5.60	3.30	2.12	4.23
This publication (ADP.P$_i$ state B)		3.75	2.27	4.06	2.97	3.94	2.93	3.51	3.39	7.82
7jy5 (chainA)	ATPγS	3.47	1.97	3.74	2.33	4.78	4.21	1.88	3.65	4.19
7lmy (chainA)	ATP	3.45	2.43	3.70	2.93	4.64	3.67	2.00	3.82	4.19
5ftn (chainA)	ATPγS	3.63	2.45	3.98	2.86	5.28	4.97	2.31	3.86	4.35
7vcv (chainA)	ATPγS	3.59	2.10	3.62	2.29	4.93	4.52	2.11	3.76	4.48
7vct (chainA)	ATPγS	3.61	2.13	3.61	2.2	4.61	4.34	2.45	3.36	5.03
7vcs (chainA)	ATPγS	3.52	2.08	3.44	2.12	3.63	3.19	2.10	3.86	5.52
7vcu (chainA)	ATPγS	3.73	2.31	5.04	3.85	5.92	4.88	3.45	2.05	4.75
7vcx (chainA)	ATPγS	3.60	2.10	3.64	2.42	4.87	4.29	2.43	3.28	4.80
7rlh (chainA)	ATPγS	3.46	2.10	3.03	2.03	4.51	4.59	2.15	3.38	4.31
7rl7 (chainA)	ATPγS	4.87	4.26	4.08	2.65	5.82	5.68	2.06	2.09	4.9
7rlj (chainA)	ATPγS	3.50	2.07	3.91	2.94	3.32	2.16	2.32	3.82	4.12
7rlc (chainA)	ATPγS	4.17	3.61	2.99	1.55	4.84	4.73	2.36	3.48	4.71
7rlf (chainA)	ATPγS	4.15	2.93	4.02	2.61	5.43	5.22	2.06	2.10	4.33
7rla (chainA)	ATPγS	3.27	2.31	4.31	3.39	5.13	4.38	2.80	3.41	5.16
5c1a (chainA)	ATPγS	3.32	2.41	4.03	2.91	4.68	3.60	2.18	2.89	4.10
3hu1 (chainA)	ATPγS	2.81	1.74	2.99	1.82	4.29	3.67	2.15	4.40	3.86
3hu2 (chainA)	ATPγS	2.98	1.81	3.06	1.86	4.29	3.99	2.20	4.25	3.50
3hu3 (chainA)	ATPγS	3.18	2.12	3.20	2.02	4.40	3.72	2.20	4.12	4.07
7lmz (chainC)	ATP	3.21	2.13	3.82	2.88	4.43	3.67	1.99	3.98	4.59
7lmz (chainD)	ATP	3.47	2.11	3.52	2.21	4.86	4.03	2.03	3.69	4.50
7lmz (chainE)	ATP	3.44	2.07	3.51	2.22	4.98	4.25	2.00	3.71	4.12
7lmz (chainF)	ATP	3.16	2.02	3.82	3.06	5.30	5.74	4.57	3.13	4.95
7ln0 (chainD)	ATP	3.47	2.05	3.91	2.81	4.72	3.72	2.02	3.75	4.21
7ln0 (chainE)	ATP	3.50	2.25	3.37	2.07	4.47	3.64	2.06	3.72	3.84
7ln0 (chainF)	ATP	3.30	2.06	3.48	2.36	4.49	3.74	4.10	3.33	4.33
7ln1 (chainD)	ATP	2.27	1.95	4.60	4.14	5.87	5.24	2.00	2.86	4.90
7ln1 (chainE)	ATP	3.28	2.04	3.19	1.92	4.29	3.5	2.02	4.15	4.33
7ln1 (chainF)	ATP	3.89	2.90	3.84	2.58	4.97	4.05	4.00	3.34	4.77
7ln2 (chainC)	ATP	2.83	1.98	2.96	1.99	4.10	3.64	2.06	4.88	4.53
7ln2 (chainD)	ATP	3.64	2.44	3.56	2.27	4.73	4.05	1.99	3.82	4.55
7ln2 (chainE)	ATP	3.23	2.04	3.32	2.2	4.53	3.76	2.23	4.13	4.81
7ln3 (chainC)	ATP	3.37	2.24	4.09	3.00	5.43	5.09	2.04	3.22	4.57
7ln3 (chainD)	ATP	3.71	2.42	3.49	2.10	4.79	4.27	2.06	3.68	4.02
7ln3 (chainE)	ATP	3.54	3.19	3.79	2.63	4.87	4.00	2.13	3.97	4.55
7ln4 (chainC)	ATP	3.22	2.36	3.34	2.65	4.31	3.50	2.28	3.90	4.35
7ln4 (chainD)	ATP	3.38	2.06	3.51	2.33	4.48	3.64	2.02	4.06	4.49
7ln4 (chainE)	ATP	3.60	2.27	3.63	2.38	4.56	3.64	2.06	4.04	4.00
7ln4 (chainF)	ATP	3.01	2.41	3.35	2.81	3.97	3.20	2.29	3.52	4.61
7ln5 (chainD)	ATP	3.34	2.07	3.63	2.67	4.37	3.46	2.01	3.88	4.27
7ln5 (chainE)	ATP	3.43	2.14	3.38	2.10	4.36	3.54	2.04	4.27	4.05
7ln5 (chainF)	ATP	2.46	2.13	3.30	3.02	4.12	3.20	2.71	4.08	4.29
7ln6 (chainD)	ATP	3.19	1.99	3.40	2.50	3.95	3.03	2.29	4.38	3.84
7ln6 (chainE)	ATP	3.67	2.74	4.21	3.62	4.68	3.76	3.44	3.79	4.01
7ln6 (chainF)	ATP	2.43	2.53	2.92	2.68	3.79	3.20	2.10	4.39	3.74

Supplementary References

1. Chen S, McMullan G, Faruqi AR, Murshudov GN, Short JM, Scheres SH*, et al.* High-resolution noise substitution to measure overfitting and validate resolution in 3D structure determination by single particle electron cryomicroscopy. *Ultramicroscopy* 2013, **135**(C)**:** 24-35.

2. Pan M, Yu Y, Ai H, Zheng Q, Xie Y, Liu L*, et al.* Mechanistic insight into substrate processing and allosteric inhibition of human p97. *Nat Struct Mol Biol* 2021, **28**(7)**:** 614-625.

3. Banerjee S, Bartesaghi A, Merk A, Rao P, Bulfer SL, Yan Y*, et al.* 2.3 A resolution cryo-EM structure of human p97 and mechanism of allosteric inhibition. *Science* 2016, **351**(6275)**:** 871-875.

4. Wendler P, Ciniawsky S, Kock M, Kube S. Structure and function of the AAA+ nucleotide binding pocket. *Biochim Biophys Acta* 2012, **1823**(1)**:** 2-14.

5. Seraphim TV, Houry WA. AAA+ proteins. *Curr Biol* 2020, **30**(6)**:** R251-R257.

6. Davis IW, Leaver-Fay A, Chen VB, Block JN, Kapral GJ, Wang X*, et al.* MolProbity: all-atom contacts and structure validation for proteins and nucleic acids. *Nucleic Acids Res* 2007, **35**(Web Server issue)**:** W375-383.

Source data for Supplementary Fig. 13

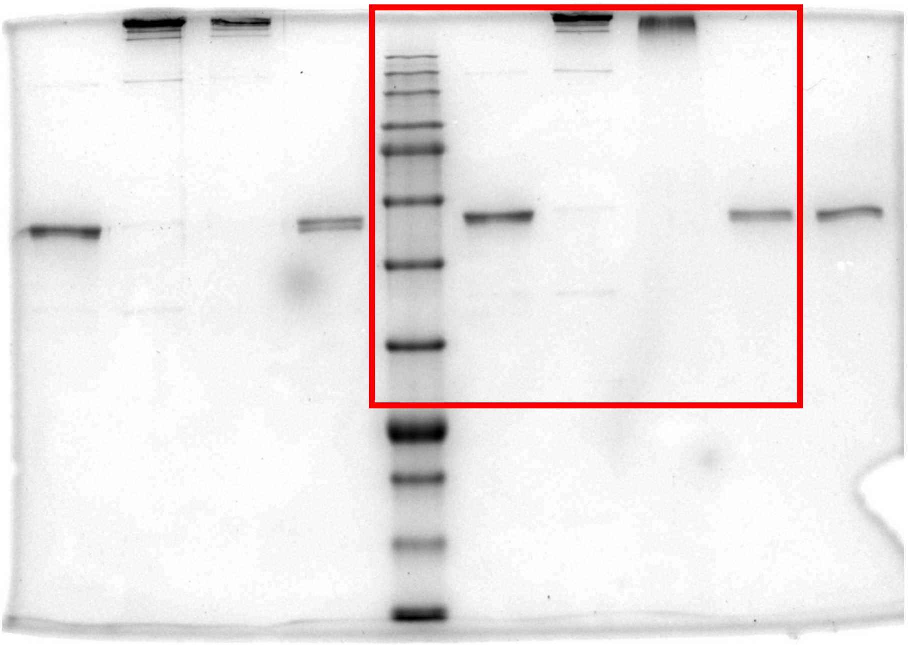

The rectangle highlights the gel area shown in Supplementary Fig. 13b.
