# Supplementary material for: Characterizing ATP processing by the AAA+ protein p97 at the atomic level
Source: Nat Chem. 2024 Feb 7;16(3):363–72. doi: 10.1038/s41557-024-01440-0 (PMC10914628; doi:10.1038/s41557-024-01440-0)
Supplement: Supplementary file 2 — Reporting Summary [file 41557_2024_1440_MOESM2_ESM.pdf]

nature portfolio

Corresponding author(s): Hitzenberger, Zacharias, Sakata, Schütz

Last updated by author(s): Nov 20, 2023

Reporting Summary

Nature Portfolio wishes to improve the reproducibility of the work that we publish. This form provides structure for consistency and transparency in reporting. For further information on Nature Portfolio policies, see our Editorial Policies and the Editorial Policy Checklist.

Statistics

For all statistical analyses, confirm that the following items are present in the figure legend, table legend, main text, or Methods section.

n/a	Confirmed	
☐	☒	The exact sample size (*n*) for each experimental group/condition, given as a discrete number and unit of measurement
☐	☒	A statement on whether measurements were taken from distinct samples or whether the same sample was measured repeatedly
☒	☐	The statistical test(s) used AND whether they are one- or two-sided<br>*Only common tests should be described solely by name; describe more complex techniques in the Methods section.*
☒	☐	A description of all covariates tested
☒	☐	A description of any assumptions or corrections, such as tests of normality and adjustment for multiple comparisons
☐	☒	A full description of the statistical parameters including central tendency (e.g. means) or other basic estimates (e.g. regression coefficient) AND variation (e.g. standard deviation) or associated estimates of uncertainty (e.g. confidence intervals)
☒	☐	For null hypothesis testing, the test statistic (e.g. *F*, *t*, *r*) with confidence intervals, effect sizes, degrees of freedom and *P* value noted<br>*Give P values as exact values whenever suitable.*
☒	☐	For Bayesian analysis, information on the choice of priors and Markov chain Monte Carlo settings
☒	☐	For hierarchical and complex designs, identification of the appropriate level for tests and full reporting of outcomes
☒	☐	Estimates of effect sizes (e.g. Cohen's *d*, Pearson's *r*), indicating how they were calculated

Our web collection on statistics for biologists contains articles on many of the points above.

Software and code

Policy information about availability of computer code

Data collection

The following software were used for cyro-EM data acquistion:
EPU v 3.1 (ThermoFisher Scientific)

The following software were used for NMR data collection:
Topspin 3.5 & 3.7 (Bruker): https://www.bruker.com/en/products-and-solutions/mr/nmr-software/topspin.html

The following software was used for MD simulations:
AMBER 2018: https://ambermd.org/
GAUSSIAN09: https://guides.libraries.uc.edu/chembio-software/gaussian

Data analysis

crYOLO 1.8.3 (Wagner et al 2019): https://cryolo.readthedocs.io/en/stable/
RELION 3.0 & 4.0 (Kimanius et al, 2021): https://relion.readthedocs.io/en/release-4.0/
MotionCor2 (Zheng et al 2017): https://emcore.ucsf.edu/ucsf-software
VMD 1.9.3 (Humphrey et al., 1996): http://www.ks.uiuc.edu/Research/vmd/
Coot (Emsley and Cowtan, 2004): http://www2.mrc-lmb.cam.ac.uk/personal/pemsley/
CTFFIND4 (Rohou and Grigorieff, 2015): http://grigoriefflab.janelia.org/ctffind4
CcpNMR 2.5.2 (CCPN): https://ccpn.ac.uk/software/version-2/
Mnova 11.0 (Mestrelab): https://mestrelab.com/software/mnova/
MicroCal PEAQ-ITC Analysis V1.21: https://www.malvernpanalytical.com/de
Uniprot 2021: https://www.uniprot.org/
UCSF Chimera 1.16 (Pettersen et al., 2004): http://www.cgl.ucsf.edu/chimera

UCSF ChimeraX 1.4 (Pettersen et al, 2021): https://www.cgl.ucsf.edu/chimerax/
GAUSSIAN09: https://guides.libraries.uc.edu/chembio-software/gaussian
CPPTRAJ (Roe and Cheatham, 2013): https://amberhub.chpc.utah.edu/cpptraj/
APBS (Jurrus et al., 2018): https://server.poissonboltzmann.org/
Clustal Omega (Sievers et al., 2011): https://www.ebi.ac.uk/Tools/msa/clustalo/
Jalview V2 (Waterhouse et al., 2009): https://www.jalview.org/
RamachanDraw: https://github.com/alxdrcirilo/RamachanDraw
Seaborn https://seaborn.pydata.org/

For manuscripts utilizing custom algorithms or software that are central to the research but not yet described in published literature, software must be made available to editors and reviewers. We strongly encourage code deposition in a community repository (e.g. GitHub). See the Nature Portfolio guidelines for submitting code & software for further information.

Data

Policy information about availability of data

All manuscripts must include a data availability statement. This statement should provide the following information, where applicable:
- Accession codes, unique identifiers, or web links for publicly available datasets
- A description of any restrictions on data availability
- For clinical datasets or third party data, please ensure that the statement adheres to our policy

Data supporting the findings of this work are available within the Article, Extended Data, the Supplementary Information and Source Data files. Further details and raw data from in-silico modelling are also available from the corresponding authors upon request. Cryo-EM maps, model coordinates and associated structure factors of p97 in ADP.Pi states have been deposited in the Electron microscopy Data Bank (EMDB code: 16781/16782) and Protein Data Bank database (PDB code: 8ooi). Publicly available datasets used can be found under PDB accession number: 3HU1, 3HU2, 3HU3, 4KO8, 5C1A, 5FTK, 5FTL, 5FTM, 5FTN, 7JY5, 7LMY, 7LMZ, 7LN0, 7LN1, 7LN2, 7LN3, 7LN4, 7LN5, 7RLA, 7RLC, 7RLF, 7RLH, 7RLJ, 7RL7, 7VCS, 7VCT, 7VCU, 7VCV, 7VCX.

Human research participants

Policy information about studies involving human research participants and Sex and Gender in Research.

Reporting on sex and gender	n.a.
Population characteristics	n.a.
Recruitment	n.a.
Ethics oversight	n.a.

Note that full information on the approval of the study protocol must also be provided in the manuscript.

Field-specific reporting

Please select the one below that is the best fit for your research. If you are not sure, read the appropriate sections before making your selection.

☒ Life sciences ☐ Behavioural & social sciences ☐ Ecological, evolutionary & environmental sciences

For a reference copy of the document with all sections, see nature.com/documents/nr-reporting-summary-flat.pdf

Life sciences study design

All studies must disclose on these points even when the disclosure is negative.

Sample size	For cryo-EM analysis, particles were collected and processed until EM maps were converged at the given resolutions. No predetermined resolution or particle number cut-offs was set for the EM analysis. Initially 10,011 images were collected and a reconstruction of 2.6 A has been obtained, which was of sufficient quality for further analysis.
Data exclusions	For cryo-EM analysis, images were inspected and rejected if considered of inadequate quality caused by ice contamination, blurriness, and bad CTF fitting. In classification process, particles in 2D and 3D classes which shows poor averages were removed.
Replication	For biochemical assays (ITC, SEC and ATPase assay), experiments were performed at least twice with consistent data. Technical replicates within experiments were minimally different. Intersubunit crosslinking experiments were performed 12 times in total, partially under slightly varied conditions. While all replication attempts were successful, the completeness of the crosslinking varied to some extent.<br><br>For cryo-EM, only one dataset was recorded.<br>For NMR, isotope labelled constructs were produced and measured only once.<br>For MD, three independent simulations of the D1 construct were performed prior to the availability of the cryo-EM data.<br>However, since all three simulations sampled slightly different ensembles with respect to the localization of the Pi ion, once the cryo-EM map

	was obtained, we were able to compare our predicted Pi positions with unassigned densities in the nucleotide binding pocket by structural alignment.<br>Additionally, we analyzed our simulation of p97 D1-D2 which was started from the preliminary cryo-EM model, which initially features all six subunits in ADP.Pi state A, but one of which shows signs of early transition to state B after ~ 1300 ns. Therefore, hallmark features that are used to define the two ADP.Pi states in the initial simulation are also found in this simulation of p97 D1-D2, confirming the initial in silico findings.
Randomization	Samples were not randomized, as mutants activities were determined by measuring fluorescent intensity compared to positive (wildtype) and negative controls.
Blinding	Blinding was not relevant to this study, as only one experimenter was performing the analysis and results were analyzed without manual scoring.

Reporting for specific materials, systems and methods

We require information from authors about some types of materials, experimental systems and methods used in many studies. Here, indicate whether each material, system or method listed is relevant to your study. If you are not sure if a list item applies to your research, read the appropriate section before selecting a response.

Materials & experimental systems

n/a	Involved in the study
☒	☐ Antibodies
☒	☐ Eukaryotic cell lines
☒	☐ Palaeontology and archaeology
☒	☐ Animals and other organisms
☒	☐ Clinical data
☒	☐ Dual use research of concern

Methods

n/a	Involved in the study
☒	☐ ChIP-seq
☒	☐ Flow cytometry
☒	☐ MRI-based neuroimaging
